# Development and validation of novel keloid-derived immortalized fibroblast cell lines

**DOI:** 10.3389/fimmu.2024.1326728

**Published:** 2024-06-10

**Authors:** Alia Sadiq, Nonhlanhla P. Khumalo, Ardeshir Bayat

**Affiliations:** MRC Wound Healing and Keloid Research Unit, Hair and Skin Research Laboratory, Division of Dermatology, Department of Medicine, Faculty of Health Sciences, Groote Schuur Hospital, University of Cape Town, Cape Town, South Africa

**Keywords:** Keloid Disease, immortalized cell line, keloid scarring, keloid fibroblast, in vitro model, h*TERT*, stable transfection

## Abstract

Keloids are a common connective tissue disorder with an ill-understood etiopathogenesis and no effective treatment. This is exacerbated because of the absence of an animal model. Patient-derived primary keloid cells are insufficient as they age through passaging and have a limited supply. Therefore, there is an unmet need for development of a cellular model that can consistently and faithfully represent keloid’s pathognomic features. In view of this, we developed keloid-derived immortalized fibroblast (KDIF) cell lines from primary keloid fibroblasts (PKF) by transfecting the human telomerase reverse transcriptase (h*TERT*) gene. The *TERT* gene encodes the catalytic subunit of the telomerase enzyme, which is responsible for maintaining the cellular replicative potential (cellular immortalization). Primary fibroblasts from keloid-specific lesional (peripheral, middle, and top) as well as extralesional sites were isolated and evaluated for cell line development and comparative cellular characteristics by employing qRT-PCR and immunofluorescence staining. Moreover, the immortalized behavior of KDIF cell lines was evaluated by comparing with cutaneous fibrosarcoma and dermatofibrosarcoma protuberans cell lines. Stable KDIF cell lines with elevated expression of h*TERT* exhibited the cellular characteristics of site-specific keloid fibroblasts. Histochemical staining for β-galactosidase revealed a significantly lower number of β-gal–positive cells in all three KDIF cell lines compared with that in PKFs. The cell growth curve pattern was studied over 10 passages for all three KDIF cell lines and was compared with the control groups. The results showed that all three KDIF cell lines grew significantly faster and obtained a fast growing characteristic as compared to primary keloid and normal fibroblasts. Phenotypic behavior in growth potential is an indication of h*TERT*-mediated immortalized transformation. Cell migration analysis revealed that the top and middle KDIF cell lines exhibited similar migration trend as site-specific PKFs. Notably, peripheral KDIF cell line showed significantly enhanced cell migration in comparison to the primary peripheral fibroblasts. All KDIF cell lines expressed Collagen I protein as a keloid-associated fibrotic marker. Functional testing with triamcinolone inhibited cell migration in KDIF. ATCC short tandem repeat profiling validated the KDIF as keloid representative cell line. In summary, we provide the first novel KDIF cell lines. These cell lines overcome the limitations related to primary cell passaging and tissue supply due to immortalized features and present an accessible and consistent experimental model for keloid research.

## 1 Introduction

Keloids are common fibroproliferative reticular dermal lesions of unknown etiopathogenesis, characterized clinically by an aggressive exophytic expansive growth into the surrounding skin with a high rate of recurrence post-therapy (1). Lack of relevant study models (including animal models as keloids only occur in human skin) (2) has been a challenging and rate-limiting issue for keloid research (3). Therefore, despite its inherent limitations compared with *in vivo* animal models, the use of *in vitro* monoculture studies using keloid-derived primary cultured fibroblasts has been invaluable in enabling studies in keloid pathobiology.

However, patient-derived primary cultured keloid fibroblasts are often scarce and insufficient as they age through passaging and have a limited supply. Additionally, keloid tissue is biologically heterogeneous with clinically distinct features within the lesion, including variable dermal cellularity, inflammatory infiltrate, and altered collagen I–III ratios (4), at margins (actively growing) compared with the center (dormant) of keloid (5), representing a variation at the cellular and molecular levels between central and peripheral keloid fibroblasts (6). This endotypic and phenotypic variation typically divides keloid into three distinct lesion-specific sites: (i) intralesional (keloid center), (ii) perilesional (keloid margin), and (iii) extralesional (normal appearing skin surrounding the lesion but distant to the margin) (7).

It has been established that *in vitro* disease–specific cell lines have lent themselves as an ideal option (a well-controlled system) to investigate the phenotypic and cellular characteristics of specific diseases. However, *in vitro* subculturing and maintenance cause aging (telomeric loss) in patient-derived primary cells (8). Significant telomeric loss was reported in keloids fibroblasts (9), resulting in a shorter lifespan and limiting the utility of keloid primary cells. Hence, induced overexpression of human telomerase reverse transcriptase (h*TERT*) has been envisioned as an attractive approach to extend lifespan of patient-derived primary cells, which is known as an “immortalized cell-line model approach.” Induced immortalization bypasses cell events like replicative senescence and cellular crisis (10, 11). Thus, these cell lines act as a gold standard as they remain genetically identical, accessible, and consistent (12). Fibroblast cells have been considered as potential candidates to develop immortalized cell line for keloid disease as they have been implicated as an important contributor to keloid pathobiology and subsequent tissue formation (13, 14).

**Figure 1:**
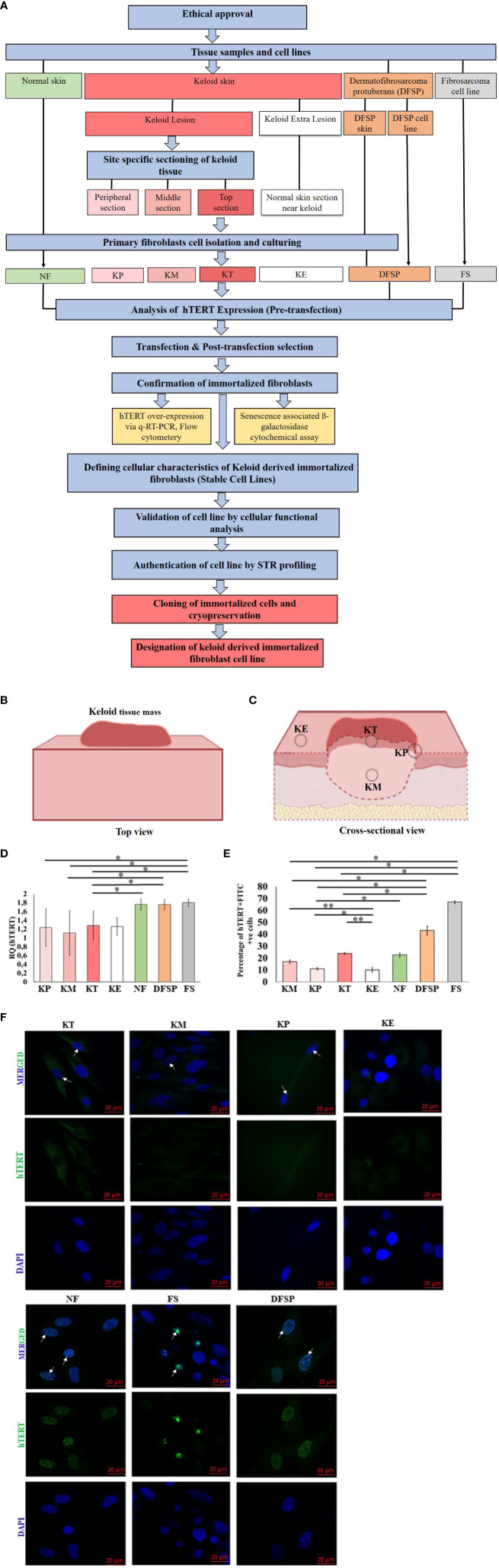
**(A)** Study methodology flow diagram. KP, keloid peripheral fibroblasts; KT, keloid top fibroblasts; KM, keloid middle fibroblasts; KE, keloid extralesional skin; NF, normal skin fibroblast; DFSP, dermatofibrosarcoma protuberans; FS, fibroblastic sarcoma cell line. **(B)** Overall bird's-eye view of keloid tissue mass. **(C)** Cross-sectional view of keloid tissue mass showing four different anatomical locations in relation to the lesion: (i) top of the keloid skin includes the superficial papillary dermis (KT), (ii) center keloid tissue includes the deep reticular dermis as middle of the keloid (KM), (iii) margin as peripheral part of keloid skin (KP), and (iv) neighboring normal appearing skin as keloid extralesional skin (KE), marked on keloid tissue mass for sectioning and isolation of site-specific primary keloid fibroblasts (PKFs). hTERT gene expression analysis in primary keloid fibroblasts (KP, KT, KM, and KE) and control groups (NF, DFSP, and FS) via **(D)** qRT-PCR and **(E)** protein expression by flow cytometry, and results were presented graphically as the percentage of hTERT-FITC–positive cells and **(F)** Immunofluorescence staining with hTERT-FITC (green, nuclear signal) and 4′,6-Diamidino-2-phenylindole dihydrochloride (DAPI) (blue, nuclear); scale bar, 20 μm. Experiments were performed in triplicate and analyzed by two-way ANOVA and significance levels set at *P < 0.05 and **P < 0.01.

In view of this, we sectioned freshly obtained biopsies of keloid tissue into four specific sites: [i] top (papillary dermis of the intralesional center of keloid), (KT) [ii] middle (reticular dermis of the intralesional center of keloid) (KM), [iii] peripheral (margin of the keloid lesion) (KP), and [iv] extralesional tissue (KE) from the same donor (**Figure 1C**). Subsequently, these site-specific keloid tissue sections were used to isolate and develop keloid-derived immortalized fibroblast (KDIF) cell lines. The present study evaluated and compared the h*TERT* expression, immortalization, and characteristics/behavior (linked with immortalization) in primary keloid fibroblasts (PKFs) before and after genetic transfection (immortalization) (KDIF), having four distinct types of control groups. The control group included (i) normal fibroblasts (NFs) isolated from normal skin tissue, donated by a non-keloid–forming participant; (ii) extralesional keloid fibroblasts (KEs), as NFs isolated from normal (in appearance) skin adjacent to the keloid tissue, donated by a keloid participant; (iii) dermatofibrosarcoma protuberans (DFSP) fibroblasts, isolated from DFSP tissue; and (iv) fibrosarcoma fibroblasts (FSs) as established cancerous cell cultures, served as positive control for h*TERT* expression. Primary fibroblast cells from the PKF, KE, and NF groups are the representative of definite lifespan (non-immortalization character), whereas primary DFSP tissue–derived fibroblast cells and FS cells are the representative of indefinite lifespan (immortalization behavior). Hence, the evaluation of immortalization behavior was crucial to define a stable immortalization in genetically transformed primary fibroblasts. Once immortalization behavior was developed and established (stable transfection), KDIF cell lines were authenticated through short tandem repeat (STR) profiling by Amercican Type Culture Collection (ATCC). To our knowledge, we provide the first authenticated and functionally validated immortalized cell line for keloid disease.

## 2 Materials and methods

Here, we provide the concise summary of the key methods and procedures employed in the current study. The detailed method and associated references are available in **SI Appendix 1**.

### 2.1 Ethical approval

Ethical approval (HREC REF Number 493/2009, 30 October 2018) of this research study (**Figures 1A–C**) was obtained from the Human Research Ethics Committee, Faculty of Health Sciences, University of Cape Town, South Africa.

### 2.2 Tissue sampling and reference cell lines

Keloid participants (N = 3) were selected as donors for keloid lesional cutaneous tissue based on the study criteria. Keloid dermal tissue was dissected into four site-specific groups: (1) peripheral (margin of the keloid) (2), middle (deep reticular component of the center of keloid) (3), top (superficial papillary component of the center of keloid), and (4) keloid extralesional skin. Normal control skin tissue (non-keloid formers with no family history of keloid disease) samples were collected from healthy female participants (N = 3) during breast reduction surgery. Skin sarcoma tissue sample was donated from cases with a clinically and histologically confirmed DFSP condition (N = 2). All skin tissue samples were obtained from participants after receiving informed ethical consent. This study also included two fibroblastic sarcoma cell lines [Fsarc-01: HT-1080 (HT1080) (ATCC® CCL-121™) and Fsarc-02: HT-1080-Luc2 (ATCC® CCL-121-LUC2™)] and one DFSP cell line (DFSP-CL) (ATCC- Hs 63T) (ATCC CRL-7043) purchased from ATCC (USA) as the reference cell lines for comparitive studies (**Supplementary Table 1**). All keloid, normal, and DFSP skin samples were processed for primary fibroblasts culturing by the collagenase method and cultured according to the standard protocol (detailed method is available in **SI Appendix 1**).

### 2.3 h*TERT* expression (pre-transfection)

The h*TERT* gene (quantitative real-time PCR) and protein expression [immunofluorescence (IF) staining and flow cytometry] were studied in PKFs before transfection and compared with the control groups.

### 2.4 h*TERT* plasmid transfection and generation of stable cell lines

An h*TERT* immortalized cell system was used to develop immortalized keloid-derived fibroblast cell line through the following steps. (1) All three groups of PKF cultures were transfected with plasmid DNA (pGRN145, MBA-141™, ATCC) containing h*TERT* gene, separately. (2) The transiently transfected cell lines were treated with hygromycin B (*HygB*; an antibiotic marker for transfection), and resistant stable cell lines were selected and cloned. (3) Analysis of h*TERT* gene transfection at mRNA level was evaluated via quantitative real-time PCR (qRT-PCR), and protein expression was evaluated by flow cytometry. (4) The validation of cellular function of hTERT protein in cell senescence was assessed by senescence-associated ß-galactosidase histochemical staining assay in all three keloid-derived transformed fibroblast cell lines.

### 2.5 Defining cellular characteristics of keloid-derived immortalized fibroblasts

The stable transfected cell lines were evaluated for (a) cell viability, (b) cell growth curve, (c) cell cycle analysis, (d) cell migration, (e) cell invasion, (f) cellular senescence, and (g) hTERT protein expression via IF staining. The KDIF cell lines were validated by testing drug (triamcinolone) sensitivity on apoptosis, viability, and cell migration.

### 2.6 Authentication of cell line: Short Tandem Repeat (STR) profiling

All three cell lines (1. PT-KT-045-stb-CL, ATCC®, Cat. No. STRB3288; 2. PT-KM-045-stb-CL, ATCC®, Cat. No. STRB3289; and 3. PT-KP-045-stb-CL, ATCC®, Cat. No. STRB3289) were authenticated by ATCC STR profiling. Successfully transformed and actively growing cell lines were selected for cloning, stock preparation, and cryopreservation.

### 2.7 Statistical analysis

All experiments were conducted in triplicate, and the results were presented graphically as mean ± standard deviation (SD), 95% confidence interval, and percentage (%) when appropriate. The statistical analysis was carried out by Student’s t-test, by using Microsoft Excel version 8. The data were also evaluated by applying two-way ANOVA and Tukey *post-hoc* test. Experiments were performed in triplicate and presented as mean ± S.D. The statistical significance level was set at *p < 0.05, **p < 0.01, and ***p < 0.001 (15).

## 3 Results

### 3.1 Primary site–specific keloid fibroblasts exhibited a comparatively low expression of h*TERT*

PKFs were isolated from four different sites (KT, KM, KP, and KE) of keloid skin tissue and then cultured and passaged three times (**Figures 1A–C**). Once cultures were established, we examined the hTERT expression in PKFs at gene (qRT-PCR) and protein levels (IF) compared with the control groups (KE, NF, DFSP, and FS). The results from h*TERT* gene expression (qRT-PCR analysis) revealed no significant difference in relative gene expression (h*TERT*) between KT, KM, KP, and KE but showed a significantly lower h*TERT* gene expression (KT, 1.28 ± 0.22, *p* < 0.023; KP, 1.24 ± 0.43, *p* < 0.04; and KM, 1.11 ± 0.51, *p* < 0.037) compared to FS (FS, 1.80 ± 0.09) (**Figure 1D**). It is also noticed that h*TERT* expression was significantly lower in KT (1.28 ± 0.22, *p* < 0.03) and KM (1.11 ± 0.51, *p* < 0.045) compared with that in DFSP (1.75 ± 0.11). A significantly low h*TERT* expression was observed in KT (1.28 ± 0.22, p < 0.03) compared with that in NF (1.76 ± 0.13).

hTERT protein expression evaluated by flow cytometry Fluorescence-Activated Cell Sorting (FACS) in KT, KM, and KP was compared with that in the control groups (KE, NF, DFSP, and FS), which revealed a significantly higher percentage of hTERT-Fluorescein isothiocyanate (FITC)–positive cell population in KT, KM, and KP (KT, 23.8 ± 0.69%, *p* < 0.0004; KP, 10.9 ± 1.03%, *p* < 0.01; and KM, 16.9 ± 1.5%, *p* < 0.0004) compared with that in KE (10 ± 1.84%) but significantly lower than that in FS (66.9 ± 1.2%, *p* < 0.003) and DFSP fibroblasts (43.1 ± 3.7%, *p* < 0.01). Furthermore, KP showed a significantly lower percentage of hTERT-FITC–positive cell population (10.9 ± 1.03%, *p* < 0.41) compared with NF (22.6 ± 1.84%) (**Figure 1E**). hTERT protein expression was also analyzed through IF analysis, and images showed no detectable nuclear signal in any of the primary keloid group compared to the control groups (**Figure 1F**).

### 3.2 Development of keloid-derived immortalized fibroblast cell lines by h*TERT* gene transfection and analysis via qRT-PCR and immunofluorescence techniques

**Figure 2:**
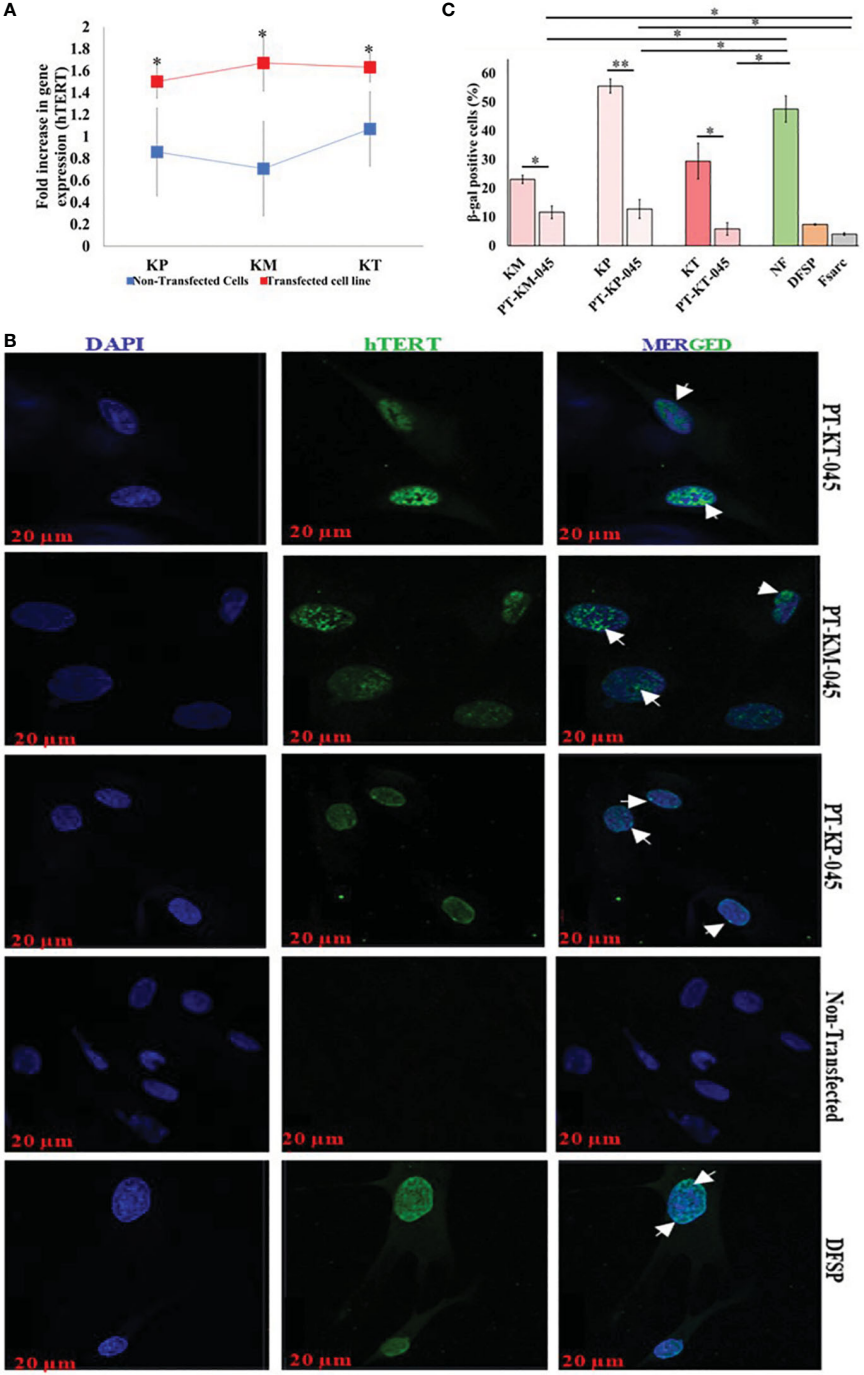
Immortalized Keloid cell line validation post-transfection. **(A)** qRT-PCR demonstrated an increase in h*TERT* gene expression (fold increase) between respective primary keloid cell lines (control, blue) and post-transfected cell lines (transfected, red). **(B)** Immunofluorescence staining with hTERT-FITC (green, nuclear signal) and DAPI (blue, nuclear); scale bar, 20 µm. **(C)** β-Galactosidase–associated senescence expressed as the percentage of stained cells and presented graphically. Experiments were performed in triplicate and presented as mean ± S.D. Significance levels were set at *P < 0.05 and **P < 0.01.

hTERT gene transfection was performed by plasmid DNA gene (*pGRN145*; carrying h*TERT* gene) transfection using GeneXPlus Transfection Reagent (Materials and Methods section, **SI Appendix 1**). Post-transfection gene expression (h*TERT*) analysis was carried out in all three transfected cell lines (PT-KT-045, PT-KM-045, and PT-KP-045). Comparative hTERT gene expression was normalized with *GAPDH* (Glyceraldehyde-3-phosphate dehydrogenase) (**Figure 2A**). The results revealed a significantly increased expression (fold change) of h*TERT* gene in all three transfected keloid cell lines (KM, 1.67 ± 0.26, *p* < 0.010; KP, 1.5 ± 0.15, *p* < 0.013; and KT, 1.63 ± 0.14, *p* < 0.027) as compared to the non-transfected PKFs (KM, 0.70 ± 0.43; KP, 0.86 ± 0.15; and KT, 1.06 ± 0.34). Post-transfected protein expression of h*TERT* gene was also evaluated by IF staining and compared with non-transfected primary keloid cells. DFSP cells were included as positive control for nuclear hTERT signal. The results showed an hTERT nuclear protein expression (green nuclear expression marked with white arrow on image) in all transfected keloid fibroblasts, compared to the non-transfected PKFs (**Figure 2B**). Moreover, post-transfection analysis of hTERT protein expression showed that PT-KT-045 cell line exhibited a significantly higher percentage of cell population expressing positive hTERT-FITC signal (28.7 ± 1.3% *p* < 0.002) (**Supplementary Figure S1**). Histochemical staining for β-galactosidase, used as a marker of cell senescence, was also evaluated in hTERT-transfected cell lines. Significantly, a lower number of β-gal–positive cells were found in all three transfected cell lines (PT-KP-045, 12.8 ± 3.30%, *p* < 0.0006; PT-KT-045, 5.89 ± 2.19%, *p* < 0.014; and PT-KM-045, 11.6 ± 0.11%, *p* < 0.002) compared to KP (55.6 ± 2.4%), KT (29.47 ± 6.2%), KM (23.10 ± 1.4%), and NF (47.6 ± 4.6%, *p* < 0.01) (**Figure 2C**). These observations suggest possible phenotypic alterations, which are likely to be linked with hTERT transfection in all three transfected keloid cell lines.

### 3.3 Selection of stably transfected cell lines, propagation, and analysis

Post-transfection screening for stably transfected cells lines was carried out by selection of HygB (concentration ranges from 0 µg/mL tp 1,000 µg/mL)–resistant cells at 48-h antibiotic treatment, through the kill curve method and cell viability evaluated by employing 3-[4,5-dimethylthiazol-2-yl]-2,5 diphenyl tetrazolium bromide (MTT) assay (**Supplementary Figures S2A–C**). The antibiotic-resistant clones were then used for studying the cellular characteristics of KDIF cell lines (**Supplementary Table 2**).

### 3.4 Cellular characteristics of keloid-derived immortalized fibroblasts

**Figure 3:**
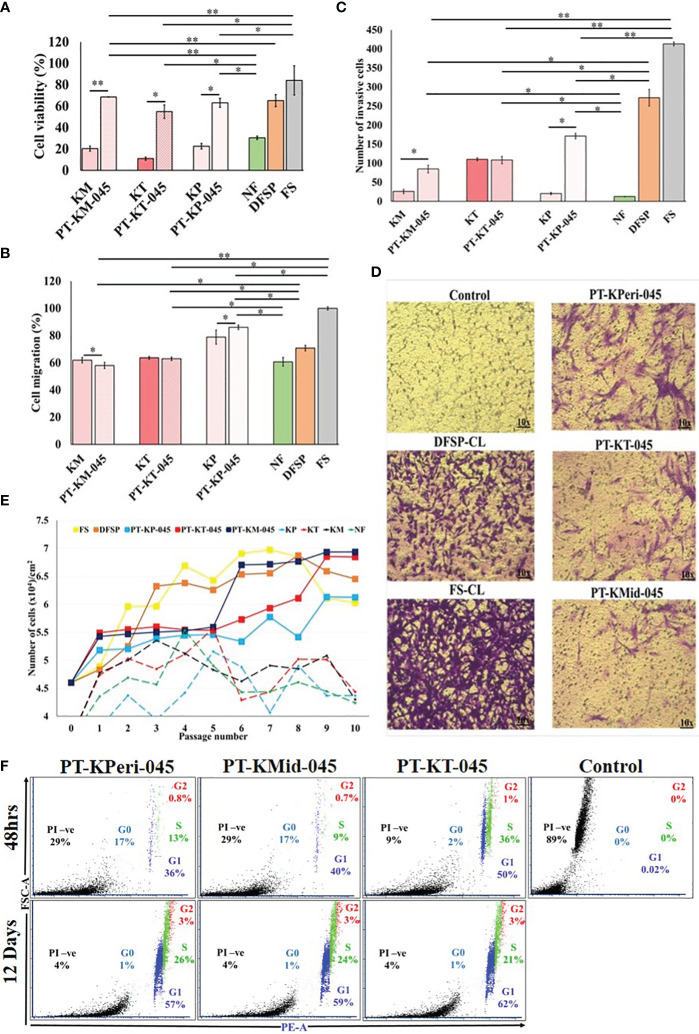
Cellular characteristics of keloid-derived immortalized fibroblast cell lines. **(A)** Cell viability, **(B)** cell migration, **(C)** cell invasion, **(D)** images showing a number of invading cells (image magnification, ×10), **(E)** population doubling assay, and **(F)** cell cycle analysis via flow cytometry using propidium iodide (PI) staining. Experiments were performed in triplicate and presented as mean ± S.D.). Significance levels were set at *P < 0.05 and **P < 0.01.

We evaluated the cellular characteristics of KDIF such as viability, growth curve, cell cycle, migration, and invasion up to 10 passage number, compared with respective PKFs. The results from MTT assay revealed that metabolic activity (cell viability) of all three KDIF cell lines significantly increased (PT-KM-045, 68.6 ± 0.28%, *p* < 2.84 E-05; PT-KP-045, 63.12 ± 4.25%, *p* < 0.013; and PT-KT-045, 54.83 ± 6.30%, *p* < 0.013) compared to the respective primary keloid cells (KM, 20.33 ± 2.4%; KT, 11.03 ± 1.56%; and KP, 22.6 ± 2.7%) and NF (30.56 ± 1.6%, *p* < 0.01). Actively viable growing behavior of KDIF cell lines exhibited similarity to DFSP and FS fibroblast growth pattern (**Figure 3A**).

Cell migration potential of PKFs has been investigated previously (16). In our study, 48-h post-scratch assay revealed that PT-KT-045 and PT-KM-045 cell lines (63.0 ± 1.3%, *p* < 0.6, and 58.07 ± 2.23%, *p* < 0.37, respectively) exhibited a similar migration trend compared to primary KT and KM fibroblasts (63.63 ± 1.04% and 61.95% ± 2.93%, respectively) except PT-KP-045 cell line that showed a significantly enhanced cell migration (86.08 ± 1.60%, *p* < 0.04) in comparison to the primary KP fibroblasts (78.8 ± 5.15%) and NF fibroblasts (60.77 ± 3.17%). Both PT-KT-045 and PT-KM-045 cell lines exhibited (63.0 ± 1.3% and 58.07 ± 2.23%, respectively) a significantly lower migration potential compared to DFSP (70.80 ± 2.02%, *p* < 0.004) and FS (100 ± 1.2%, *p* < 0.0003) (**Figure 3B**; **Supplementary Figure S3**).

hTERT overexpression is known to activate invasive behavior in cells (17); therefore, we investigated the cellular invasive potential in all three KDIF cell lines. It was noticed that number of invasive cells were significantly higher in all KDIF cell lines (PT-KP-045, 171 ± 7.5, *p* <0.001, PT-KM-045, 85 ± 10.3, *p* < 0.003 and PT-KT-045, 108.5 ± 9.8, *p* < 0.007) compared to NF (12.5±0.57). Notably, Both PT-KM-045 and PT-KP-045 cell lines showed significantly (85 ± 10.3, *p* < 0.029; 171 ± 7.5, *p* < 0.002, respectively) increased directional response (chemotaxis) towards growth factor fetal bovine serum (FBS), that resulted in their ability to migrate through a physical barrier towards chemo-attractant gradient, as compared to the primary KM and KP fibroblasts (26 ± 5.7 and 20.5 ± 2.9, respectively). Thus, hTERT overexpression in KDIF cell lines enhanced the cellular chemotaxis and directional response, indicating a similarity in behavior with DFSP and FS (**Figures 3C, D**).

The cell growth curve pattern had been studied over 10 passages for all three KDIF cell lines and compared with control groups. The results showed that all three KDIF cell lines grew significantly faster and obtained a fast growing characteristic at passage 9 (**Figure 3E**) compared to primary keloid and NFs. This phenotypic behavior in growth potential indicated the result of spontaneous hTERT-mediated immortalized transformation. Observations about cell viability and growth curve were further investigated by quantification of total DNA content at the G0, G1, S, and G2 phases of cell cycle in all three cell line’s populations at 48-h and 12-day time points. Among all KDIF cell lines, PT-KT-045 cell line showed the highest percentage of cell population at the G1 phase (growth phase) (51.51 ± 0.23% *p* < 0.016) and at the S phase (36.15 ± 1.23% *p* < 0.002), at 48-h time point, that increased up to (83 ± 1.22% *p* < 0.001) the 12-day time point (**Figure 3F**), representing a fast growing cell line during early and late culture time points, which shows its active cell growth potential throughout the cell culturing.

### 3.5 Validation of keloid-derived immortalized fibroblast cell lines

**Figure 4:**
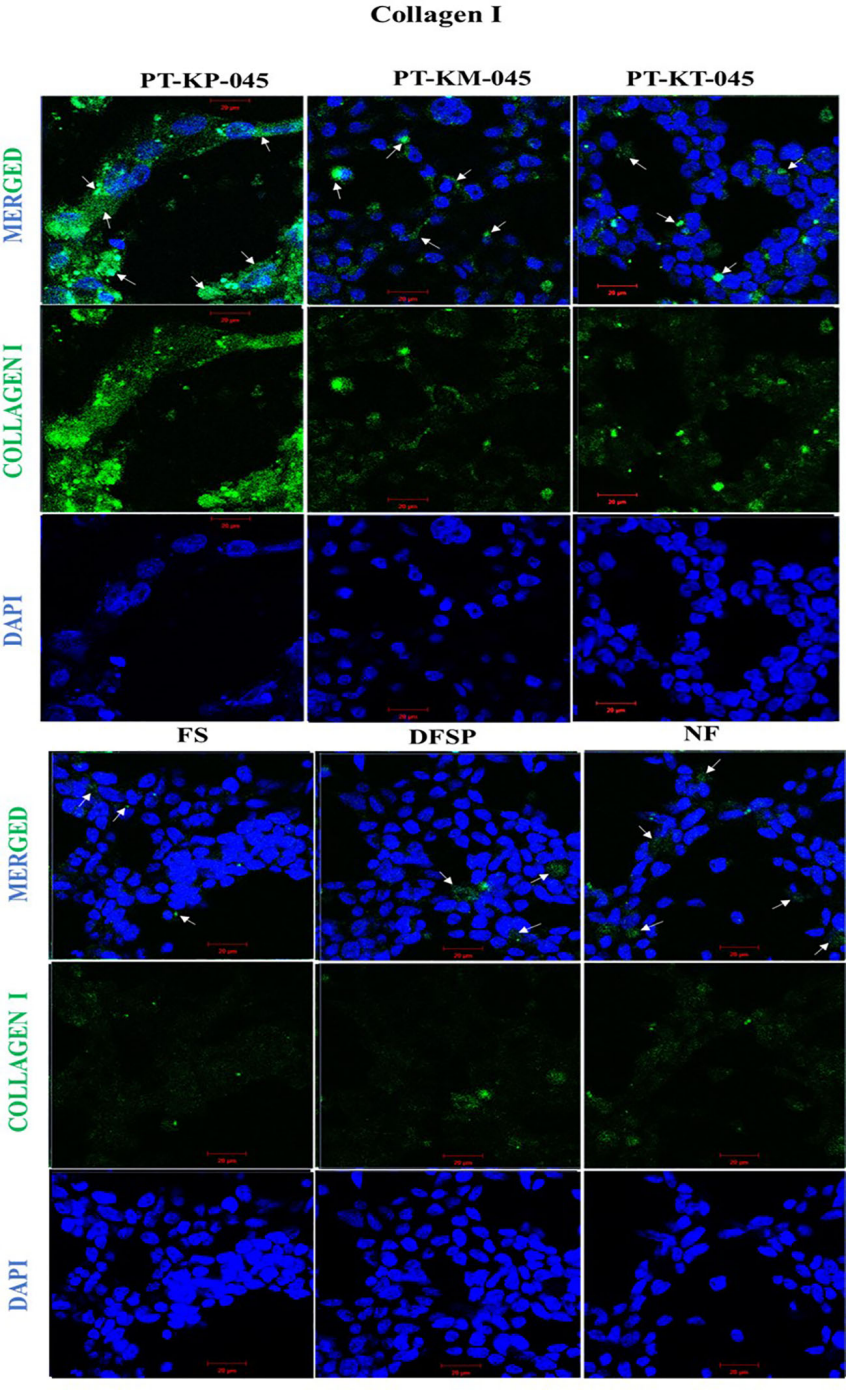
Immunofluorescence staining of increased collagen I protein expression in keloid cell lines compared with control. Cells were immunofluorescent labeled with anti-rabbit Alexa 488 (green, signal) marked with white arrows, and counterstained with DAPI (blue, nuclear signal) in transfected keloid fibroblasts group (PT-KP-045, PT-KT-045, and PT-KM-045) and DFSP, FS, and NF included as +ve control group. Scale bar set at 20 µm.

The immortalization of cells, through overexpression of telomerase (hTERT), has the advantage of maintaining a stable cellular phenotype as the cells remain diploid, but primary cultures from independent isolations can vary. Their overall growth characteristic and cellular response can also be altered due to introduced changes in their genetic makeup during the process of immortalization (18). Therefore, it is necessary to investigate and compare the cellular response of KDIF cell lines to see whether these cell lines represent keloid cell behavior or characteristics that would further validate their candidature as a representative model. Therefore, we studied the protein expression of collagen I in all three KDIF cell lines via IF staining. All three KDIF cell lines showed an increased protein expression of collagen I (**Figure 4**).

### 3.6 Functional validation: triamcinolone drug treatment decreased the cell viability and cell migration in KDIF and PKF cell lines

**Figure 5:**
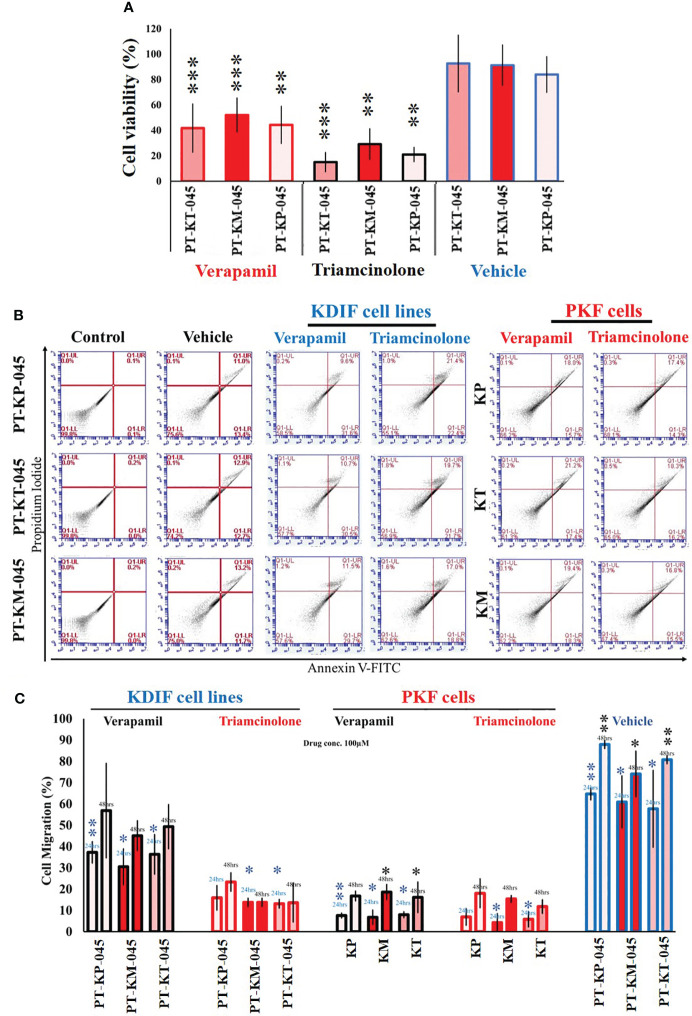
Functional validation of Keloid cell lines with drug testing. **(A)** Effect of verapamil and triamcinolone drug treatment (100 µM each) evaluated on cell viability of KDIF (PT-KT-045, PT-KM-045, and PT-KP-045) and percentage (%) cell viability was significantly decreased compared to vehicle group (untreated). **(B)** Effect of verapamil and triamcinolone drug treatment (100 µM each) evaluated on cellular apoptosis in KDIF, detected by staining with annexin V–conjugated FITC through flow cytometry. **(C)** Effects of verapamil and triamcinolone on cell migration represented graphically as the percentage (%) cell migration toward scratch zone at 24-h and 48-h time points. Experiments were performed in triplicate and presented as mean ± S.D.). Significance levels were set at *p < 0.05, **p < 0.01 and ***p < 0.001.

Studies have shown that triamcinolone and verapamil affect cell viability and induce apoptosis in PKFs (19–21) and are used clinically to treat keloids (22). Hence, KDIF cell lines were subjected to functional analysis and drug response studies for verapamil and triamcinolone. KDIF cell lines showed sensitivity to triamcinolone (100 µM each), which decreased cell viability significantly up to 20 ± 1.3% (*p* < 0.02) in all KDIF cell lines compared to the control group (untreated) (**Figure 5A**). Further investigation using annexin V apoptotic marker through flow cytometry analysis showed an increased percentage of cell population, positive for pre-apoptotic signal (Annexin V), in response to verapamil and, particularly, triamcinolone drug treatment (100 µM each) in KDIF (PT-KP-045, 22.4 ± 1.2%, *p* < 0.001; PT-KT-045, 21.7 ± 0.6%, *p* < 0.003; and PT-KM-045, 18.8 ± 0.7%, *p* < 0.03) as well as PKF cell line group (KP, 14.3 ± 0.2%, *p* < 0.006; KT, 16.2 ± 0.04%, *p* < 0.01; and KM, 15.5 ± 0.3%, *p* < 0.025) compared to the vehicle group (**Figure 5B**). These results confirmed the cellular response of KDIF cell lines toward drug treatment and represent PKF cell line’s drug sensitivity particularly for triamcinolone. Additionally, the effects of verapamil and triamcinolone (100 µM) on cell migration of KDIF and PKF cell lines were evaluated by employing an *in vitro* scratch assay at 24-h and 48-h time points. Triamcinolone significantly reduced the cell migration rate at both time points in all KDIF (PT-KP-045, 23.38 ± 4.50%, *p* < 0.0002; PT-KT-045, 13.64 ± 9.2%, *p* < 0.004; and PT-KM-045, 13.82 ± 2.11%, *p* < 0.009) as well as PKF cell lines (KP, 18.08 ± 6.9%, *p* < 0.001; KT, 11.84 ± 3.4%, *p* > 0.7; and KM, 15.40 ± 1.8%, *p* < 0.009) (**Figure 5C**), providing another supportive information about the representativeness of KDIF in terms of keloid behavior.

### 3.7 Authentication and designation

**Figure 6:**
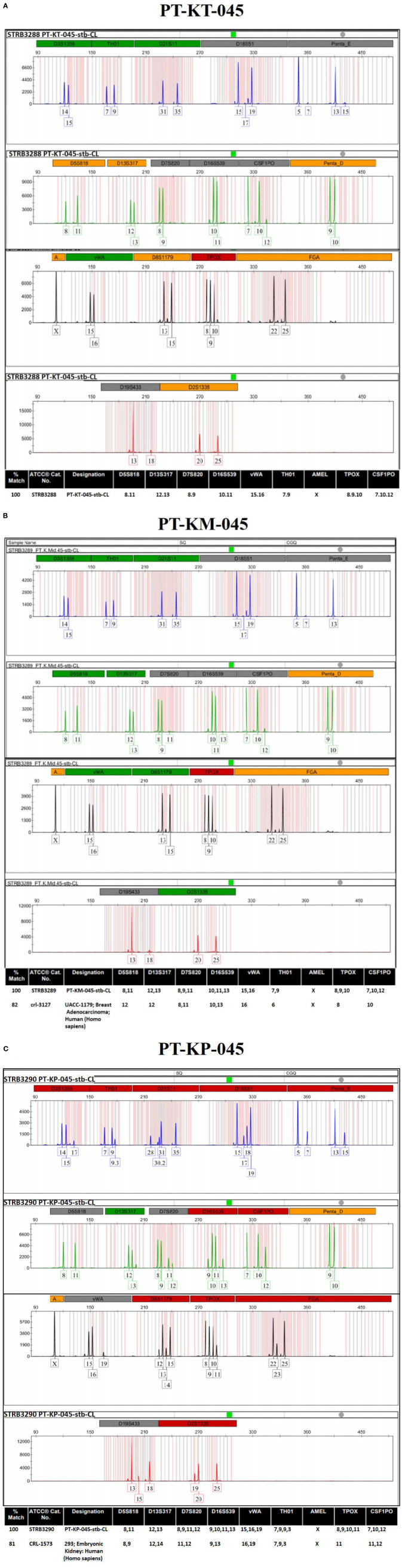
ATCC authentication of keloid-derived immortalized fibroblast cell lines via STR profiling. Electropherogram showing the 17 most repetitive polymorphic markers plus Amelogenin gene (D3S1358, TH01, D21S11, D18S51, Penta_E, D5S818, D13S317, D7S820, D16S539, CSF1PO, Penta_D, Amelogenin, vWA, D8S1179, TPOX, FGA, D19S433, and D2S1338) to develop the unique STR identity for **(A)** PT-KT-045, **(B)** PT-KM-045, and **(C)** PT-KP-045 human cell lines.

After functional validation, cell line identity and purity were evaluated for all three KDIF cell lines, by using standard genotyping technique such as the gold standard, STR profiling through ATCC services for human cell lines. Human cell line authentication assay identifies STR markers, which are short repetitive segments of DNA found between genes, at specific loci to established DNA fingerprints for every human cell line. This process involves PCR amplification of 17 most repetitive polymorphic markers plus Amelogenin gene in human genome and pattern use to develop unique identity profile of human cell lines. The results were presented as electropherogram showing the highest matches to the sample profile in the database along with the standard loci for each submitted cell line. First immortalized cell line labeled “PT-KT-045,” derived from primary “Keloid Top fibroblasts (KT)” (ATCC^®^, Cat. No. STRB3288) (**Figure 6A**), shows no match with ATCC database at any of the Loci and is designated as “PT-KT-045-stb-CL” having unique STR identity. Electropherogram for the second immortalized cell line labeled “PT-KM-045,” derived from primary “Keloid Middle fibroblasts (KM),” is designated as “PT-KM-045-stb-CL” (ATCC®, Cat. No. STRB3289). It is showing 100% match for unique STR profile and 82% relatedness with UACC-1197 Breast Adenocarcinoma (crl-3127) from ATCC database (*Homo sapiens*) (**Figure 6B**). Third immortalized cell line labeled “PT-KP-045,” derived from primary “Keloid Peripheral fibroblasts (KP),” designated as “PT-KP-045-stb-CL,” (ATCC®, Cat. No. STRB3289), shows 100% match for unique STR profile and 81% relatedness with 293 embryonic kidney cells (*Homo Sapiens*) (CRL-1573) from ATCC database (**Figure 6C**). All results based on STR profiling confirmed that all three KDIF cell lines are authenticated as novel, clean, and originated from the same human source (female) (100% match), karyotypically normal and not contaminated with any other source.

## 4 Discussion

Lack of a relevant study model and the absence of an animal model pose a challenge in studying keloid pathobiology. In addition, patient-derived cultured PKFs are sub-optimal as they are heterogeneous in nature depending on lesional site of origin and have a limited lifespan as they age through passaging with a limited tissue supply. Thus, there is an unmet need to develop a robust and relevant cellular model that can faithfully represent keloid’s pathognomic features.

Thus, we present, for the first time, KDIF cell lines from primary fibroblasts obtained from specific lesional sites (peripheral, middle, and top) within keloid tissue. We employed the strategy of overexpressing the h*TERT* gene to support the immortalized characteristic phenotype in keloid cell lines. For this purpose, specifically, we used the non-viral plasmid vector containing h*TERT* gene (pGRN145) as compared to available viral vectors (i.e., SV40), to eliminate any possibility of viral contamination in the genome of keloid fibroblast and to prevent the potential emergence of a cancerous phenotype in these cells. This approach was developed on the basis of the observation/knowledge that keloids exhibit a distinct nature compared to malignant tumors, which is clearly shown in our study by including two control groups of soft tissue carcinomas (1): DFSP and (2) FS. Through experiments utilizing qRT-PCR, flow cytometry, and IF techniques, we observed that the expression of the h*TERT* gene and protein in all three keloid site-specific primary fibroblasts (KT, KM, and KP) was lower when compared to DFSP and FS (as shown in **Figures 1D–F**).

Low hTERT expression in all PKF is responsible for limited lifespan in primary fibroblasts that can be counteracted by increased activity of telomerase, thus preserving telomere length and cellular functions (23). Hence, we envisioned this approach to extend the lifespan of patient-derived primary cells, which is known as the “immortalized cell line model approach” in order to induce overexpression of hTERT. Subsequently, we developed a KDIF cell line by introducing h*TERT* gene via transfection in all three site-specific PKF cells (peripheral, middle, and top keloid fibroblasts). Additionally, we demonstrated that stable KDIF cell lines showed enhanced expression of hTERT. The introduction of the h*TERT* gene is a widely used strategy to extend the lifespan of many cell types, and successful immortalization has, for instance, previously been reported in human retinal pigment epithelial cells (24).

Reconstitution of telomerase activity by induced expression of hTERT results in an immortal phenotype in various types of normal human cells, including fibroblasts. Despite transformation characteristics, it is unclear whether hTERT-immortalized cells are physiologically and biochemically the same as their normal counterparts (25). In view of the fact that continuous cell expansion always provides a selective advantage for rapid growth, the cellular phenotype can occasionally become biased because of the overgrowth of the most rapidly dividing cells, rather than the best differentiating cells (18). Therefore, we also evaluated the cellular as well as the functional characteristics of KDIF cell lines. Significantly improved cell viability and cell growth curve showed a fast growing trend in all three KDIF cell lines as a result of spontaneous hTERT-mediated immortal transformation. Observations about cell viability and growth curve were further evaluated by studying the cell cycle phase, in all three cell lines at 48-h and 12-day time point by flow cytometry. PT-KP-045 cell line was noticed as actively growing cells at the late stage of culturing and surpassing senescence. Furthermore, it was observed that all KDIF cell lines exhibited decreased senescence activity. Moreover, KDIF cell lines exhibited significantly increased cell migration and invasion, owing to hTERT overexpression, which is known to promote cell migration (16, 26, 27).

It is well recognized that increased Collagen I protein expression is associated with dermal fibrosis (28). Previous studies reported a significantly elevated protein expression of collagen 1 in keloid fibroblasts particularly at the growing margins of keloid scars (4). In this study, IF microscopy showed the increased expression of collagen 1 protein in every KDIF cell line, with notably abundant presence in PT-KP-045 cell line. Even though our preliminary testing on collagen 1 expression appears limited; this was conducted in order to provide additional insight into functional evaluation of our preliminary findings.

Furthermore, the use of triamcinolone for treating the keloid fibroblasts has been an active area of research (29–31), as it has shown effectiveness in modulating fibroblast activity (32). In our functional testing of drug efficacy on immortalized keloid fibroblast cell line, we specifically investigated that triamcinolone treatment–induced apoptosis affected viability and inhibited cell migration in KDIF cell lines as these are primary and core cellular characteristics identified during the fibroproliferative phenotypic development of keloids. These outcomes validate the responsiveness of KDIF cells to the drug and highlight the particular sensitivity of PKF cells to triamcinolone. This functional validation provides additional information about the representativeness of PKF as a suitable model for studying the cellular and therapeutic response of keloid fibroblasts in an *in vitro* research setting.

Moreover, human cell line identity and purity determination by “STR profiling” for all three KDIF cell lines established KDIF cell lines akin to standard cell lines exempted from genetic variation (genetically identical populations) and provided an unlimited cell population that overcomes the problem commonly encountered with limited supply of PKFs. In summary, these results demonstrate evident genetic, cellular, and biological alterations (h*TERT* gene–derived immortalization) in all KDIF cell lines as they represent keloid cellular behavior and characteristics, confirming their candidature as a suitable in vitro model for research into keloids.

## Data availability statement

The original contributions presented in the study are included in the article/**Supplementary Material**. Further inquiries can be directed to the corresponding author.

## Ethics statement

The studies involving humans were approved by Human Research Ethics Committee (HREC REF Number 493/2009, Date 30/10/2018), Faculty of Health Sciences,University of Cape Town, South Africa. The studies were conducted in accordance with the local ethical guidelines and institutional requirements. The participants provided their written informed consent to participate in this study.

## Author contributions

AS: Data curation, Formal analysis, Investigation, Methodology, Validation, Visualization, Writing – original draft, Writing – review & editing. AB: Conceptualization, Formal analysis, Funding acquisition, Investigation, Methodology, Project administration, Resources, Supervision, Validation, Visualization, Writing – review & editing. NPK: Formal analysis, Funding acquisition, Investigation, Methodology, Project administration, Resources, Supervision, Validation, Visualization, Writing – review & editing.

## References

[1] Bayat A Arscott G Ollier WER Ferguson MWJ McGrouther DA. 'Aggressive keloid': A severe variant of familial keloid scarring. J R Soc Med. (2003) 96:554–5. doi: 10.1258/jrsm.96.11.554 PMC539632 14594967

[2] Sharma JR Lebeko M Kidzeru EB Khumalo NP Bayat A. *In vitro* and ex vivo models for functional testing of therapeutic anti-scarring drug targets in keloids. Adv Wound Care (New Rochelle). (2019) 8:655–70. doi: 10.1089/wound.2019.1040 PMC6904937 31827980

[3] Ud-Din S Thomas G Morris J Bayat A. Photodynamic therapy: an innovative approach to the treatment of keloid disease evaluated using subjective and objective non-invasive tools. Arch Dermatol Res. (2013) 305:205–14. doi: 10.1007/s00403-012-1295-4 23117184

[4] Syed F Ahmadi E Iqbal SA Singh S McGrouther DA Bayat A. Fibroblasts from the growing margin of keloid scars produce higher levels of collagen I and III compared with intralesional and extralesional sites: clinical implications for lesional site-directed therapy. Br J Dermatol. (2011) 164:83–96. doi: 10.1111/bjd.2010.164.issue-1 20849516

[5] Bayat A Arscott G Ollier WER Fergusond MWJ McGrouthera DA. Description of site-specific morphology of keloid phenotypes in an Afrocaribbean population. J Plast Reconstr Aesthet Surg. (2004) 57:122–33. doi: 10.1016/j.bjps.2003.11.009 15037166

[6] Jumper N Paus R Bayat A. Functional histopathology of keloid disease. Histol Histopathol. (2015) 30:1033–57. doi: 10.14670/HH-11-624 25900252

[7] Bagabir R Syed F Paus R Bayat A. Long-term organ culture of keloid disease tissue. Exp Dermatol. (2012) 21:376–81. doi: 10.1111/j.1600-0625.2012.01476.x 22509836

[8] Caley M Wall IB Peake M Kipling D Giles P Thomas DW. Development and characterisation of a Human Chronic Skin Wound Cell Line-Towards an alternative for animal Experimentation. Int J Mol Sci. (2018) 19:1001. doi: 10.3390/ijms19041001 29584680 PMC5979489

[9] De Felice B Wilson RR Nacca M. Telomere shortening may be associated with human keloids. BMC Med Genet. (2009) 10:110. doi: 10.1186/1471-2350-10-110 19863817 PMC2774319

[10] Kassem M Abdallah BM Yu Z Ditzel N Burns JS. The use of hTERT-immortalized cells in tissue engineering. Cytotechnology. (2004) 45:39–46. doi: 10.1007/s10616-004-5124-2 19003242 PMC3449958

[11] Cukusic A Vidacek NS Sopta M Rubelj I. Telomerase regulation at the crossroads of cell fate. Cytogenet Genome Res. (2008) 122:263–72. doi: 10.1159/000167812 19188695

[12] Niu N Wang L. *In vitro* human cell line models to predict clinical response to anticancer drugs. Pharmacogenomics. (2015) 16:273–85. doi: 10.2217/pgs.14.170 PMC4358765 25712190

[13] Zhang X Yin M Zhang LJ. Keratin 6, 16 and 17-critical barrier alarmin molecules in skin wounds and psoriasis. Cells. (2019) 8:807. doi: 10.3390/cells8080807 31374826 PMC6721482

[14] Nangole FW Agak GW. Keloid pathophysiology: fibroblast or inflammatory disorders? JPRAS Open. (2019) 22:44–54. doi: 10.1016/j.jpra.2019.09.004 32051841 PMC7015170

[15] Sadiq A Shah A Jeschke MG Belo C Hayat MQ Murad S. The role of serotonin during skin healing in post-thermal injury. Int J Mol Sci. (2018) 19:1034. doi: 10.3390/ijms19041034 29596386 PMC5979562

[16] Kim H Anggradita LD Lee S-J Hur SS Bae J Hwang NS-Y. Ameliorating fibrotic phenotypes of keloid dermal fibroblasts through an epidermal growth factor-mediated extracellular matrix remodeling. Int J Mol Sci. (2021) 22:2198. doi: 10.3390/ijms22042198 33672186 PMC7926382

[17] Park Y-J Kim EK Bae JY Moon S Kim J. Human telomerase reverse transcriptase (hTERT) promotes cancer invasion by modulating cathepsin D *via* early growth response (EGR)-1. Cancer Lett. (2016) 370:222–31. doi: 10.1016/j.canlet.2015.10.021 26519755

[18] Robin JD Wright WE Zou Y Cossette SC Lawlor MW Gussoni E. Isolation and immortalization of patient-derived cell lines from muscle biopsy for disease modelling. J Vis Exp. (2015) 95:e52307. doi: 10.3791/52307-v PMC4354544 25651101

[19] Giugliano G Pasqualib D Notarob A Brongoa S Nicolettia G D’Andreaa F. Verapamil inhibits interleukin-6 and vascular endothelial growth factor production in primary cultures of keloid fibroblasts. Br Assoc Plas Surg. (2003) 56:804–9. doi: 10.1016/S0007-1226(03)00384-9 14615256

[20] Chen AD Chen RF Li YT Huang YT Lin SD Lai CS. Triamcinolone acetonide suppresses keloid formation through enhancing apoptosis in a nude mouse model. Ann Plast Surg. (2019) 83:S50–4. doi: 10.1097/SAP.0000000000002090 31513066

[21] Euler T Valesky EM Meissner M Hrgovic I Kaufmann R Kippenberger S. Normal and keloid fibroblasts are differentially influenced by IFN-γ and triamcinolone as well as by their combination. Wound Repair Regener. (2019) 27:450–61. doi: 10.1111/wrr.12722 30994217

[22] Kara K Richard S. Treatment of keloids: A meta-analysis of intralesional triamcinolone, verapamil, and their combination. Plast Reconstruct Surg. (2022) 10:e4075. doi: 10.1097/GOX.0000000000004075 PMC8849409 35186630

[23] Vaiserman A Krasnienkov D. Telomere length as a marker of biological age: state-of-the-art, open issues, and future perspectives. Front Genet. (2021) 11:630186. doi: 10.3389/fgene.2020.630186 33552142 PMC7859450

[24] Su F Liu X Liu G Yu Y Wang Y Yaping J. Establishment and evaluation of a stable cattle type II alveolar epithelial cell line. PloS One. (2013) 8:e76036. doi: 10.1371/journal.pone.0076036 24086682 PMC3784436

[25] Lindvall C Hou M Komurasaki T Zheng C Henriksson M Sedivy JM. Molecular characterization of human telomerase reverse transcriptase-immortalized human fibroblasts by gene expression profiling: activation of the epiregulin gene. Cancer Res. (2003) 63:1743–7. 12702554

[26] Liu H Liu Q Ge Y Zhao Q Zheng X Zhao Y. hTERT promotes cell adhesion and migration independent of telomerase activity. Sci Rep. (2016) 6:22886. doi: 10.1038/srep22886 26971878 PMC4789728

[27] Liu Q Wang X Jia Y Long X Yu N Wang Y. Increased blood flow in keloids and adjacent skin revealed by laser speckle contrast imaging. Lasers Surg Med. (2016) 48:360–4. doi: 10.1002/lsm.22470 26749479

[28] Ashcroft KJ Syed F Bayat A. Site-specific keloid fibroblasts alter the behaviour of normal skin and normal scar fibroblasts through paracrine signalling. PloS One. (2013) 8:e75600. doi: 10.1371/journal.pone.0075600 24348987 PMC3857170

[29] McCoy BJ Diegelmann RF Cohen IK. *In vitro* inhibition of cell growth, collagen synthesis, and prolyl hydroxylase activity by triamcinolone acetonide. Proc Soc Exp Biol Med. (1980) 163:216–22. doi: 10.3181/00379727-163-40750 6244596

[30] Oddera S Cagnoni F Mangraviti S Giron-Michel J Popova O Canonica GW. Effects of triamcinolone acetonide on adult human lung fibroblasts: decrease in proliferation, surface molecule expression and mediator release. Int Arch Allergy Immunol. (2002) 129:152–9. doi: 10.1159/000065877 12403933

[31] Yang TH Gingery A Thoreson AR Larson DR Zhao C Amadio PC. Triamcinolone Acetonide affects TGF-β signaling regulation of fibrosis in idiopathic carpal tunnel syndrome. BMC Musculoskelet Disord. (2018) 19:342. doi: 10.1186/s12891-018-2260-y 30243295 PMC6151186

[32] Johnson BZ Stevenson AW Prêle CM Fear MW Wood FM. The role of IL-6 in skin fibrosis and cutaneous wound healing. Biomedicines. (2020) 8:101. doi: 10.3390/biomedicines8050101 32365896 PMC7277690

