# Supplementary material for: Development and validation of novel keloid-derived immortalized fibroblast cell lines
Source: Front Immunol. 2024 Jun 10;15:1326728. doi: 10.3389/fimmu.2024.1326728 (PMC11194733; doi:10.3389/fimmu.2024.1326728)
Supplement: Supplementary file 1 [file DataSheet_1.pdf]

Development & Validation of Novel Keloid-Derived Immortalized Fibroblast Cell Lines

Alia Sadiq, Nonhlanhla P Khumalo, Ardeshir Bayat

SI Appendix 1.

I. **Supporting Material & Methods**

II. **Supplementary Figures and Tables**

III. **Supporting Information References**

Supporting Material & Methods

Tissue sampling and Reference cell lines

Keloid participants (N=3) were selected as donor for keloid skin tissue according to the study criteria, derived from literature [1-5]**.** The study criteria consist of (1) Participants should not have any other health condition except keloid, (2) Participant's age group should ranges between 18-65 years**,** (3) None of the participant should be smokers (4) None of the participant should be receiving any medication. Based on this criteria age, gender and keloid site-matched participants were selected for keloid tissue sampling. Keloid skin excisional Biopsy (4 cm) from (i) top of the keloid skin includes the superficial papillary dermis (KT), (ii) center keloid tissue includes the deep reticular dermis as middle of the keloid (KM), (iii) margin as peripheral part of keloid skin (KP), and (iv) neighbouring normal appearing skin as keloid extralesional skin (KE) for comparison. In general, two skin biopsies (4mm punch) were obtained from each site. Control groups were consisted of normal skin tissue and soft tissue sarcoma (skin). Normal skin tissue was donated by normal healthy female participants (N=3), during breast reduction surgery and skin sarcoma tissue sample was donated by participants exhibiting Dermatofibrosarcoma protuberans (DFSP) condition (N=2). All skin tissue samples were obtained from participants after getting informed consent. This study also included two Fibroblastic sarcoma cell lines, (Fsarc-01: HT-1080 [HT1080] (ATCC® CCL-121™; Fsarc-02: HT-1080-Luc2 (ATCC® CCL-121-LUC2™) and one Dermatofibrosarcoma protuberans cell line (DFSP-CL) (ATCC- Hs 63T (ATCC CRL-7043) purchased from American Type Culture Collection (ATCC, USA) as reference cell lines for comparitive studies (Supplementary Table. 1).

Isolation of Primary Keloid Fibroblasts (PKF) and subculturing

Keloid tissue samples dissected into four site specific groups (1) Peripheral section, (2) Middle section, (3) Top section, (4) Keloid Extralesional skin section from distant neighboring keloid margins. Experimental group include primary keloid fibroblasts isolated from peripheral, middle and transitional sites of whole keloid tissue mass and primary normal dermal fibroblasts isolated from neighboring normal keloid dermal skin. Control group included primary normal dermal fibroblasts isolated from breast reduction leftover surgical skin tissue samples and primary dermal fibroblasts isolated from Dermatofibrosarcoma protuberans skin tissue samples.

All keloid, normal and DFSP skin samples were processed for primary fibroblasts culturing by collagenase method which is adopted and modified from Burn Research and Skin Regeneration Lab, Sunnybrook Research Institute, Toronto, ON, Canada. Collagenase cocktail was consisted of 20X dispase 5 ml (1X), 0.25% trypsin 20 ml (0.05%), 1000 U/ml collagenase (1000U; amount of collagenase was calculated according to the units on the bottle, i: e [(1000 U/ml) * 100 ml] / 125 U/mg = 800 mg collagenase in 100 ml collagenase cocktail), DMEM with 1% AB/AM 75 ml. After preparing collagenase cocktail, mini incubator was turned on and used for mixing up collagenase cocktail. Collagenase cocktail was filtered through 100 µm filter into 50 mL tubes (need 10-20 ml per tube depending on the amount of sample). Cocktail kept warmed while processing the tissue. Tissue processing; first a few drops of PBS and P/S added into petri dish and tissue was placed onto these drops. The epidermis was removed with the help of blade and forceps, horizontally. Tissue was chopped as fine as possible with sterile scissors or scalpel (looked like oatmeal but finer chopped). Tissue drying off was avoided during processing by adding PBS (a few drops). Once tissue was chopped finely, collagenase cocktail was added, and placed the 50 ml falcon containing tissue cocktail in mini incubator for 30 min at 37 degrees for tissue digestion. Syringe (20 mL) was

used to draw the cocktail up and down, trying to break up any large pieces and again put into mini incubator for 20 min at 37 degrees. After this, tissue digestion test was performed by using a 20 mL syringe with 16G needle to draw the cocktail up and down. Once it passed through the 16G needle smoothly, cocktail was poured through 100 µm filter into another 50 mL tube and spin down at 1000 rpm for 5-10 min. After pelleting down, supernatant was removed by suction and resuspended in cell growth complete media [DMEM 500 ml (Gibco), 10% FBS 50 ml, 1% AB/AM 5 ml] for primary fibroblasts cell culturing and plated down in tissue culture flask (T75) with appropriate labelling. Culture flasks were kept in humidified incubator (37°C, 95% O2, 5% CO2) for culturing, until a few days (3-5days) to establish primary culture, and afterwards, culture medium was refreshed every 3 days. Spindle-like proliferating cells were observed as fibroblasts in all study groups (KP; Keloid Peripheral fibroblasts, KT; Keloid Top fibroblasts, KM; Keloid Middle fibroblasts, KE; Keloid Extralesional skin fibroblasts, NF; Normal fibroblast, DFSP; Dermatofibrosarcoma protuberans), under microscope. The confluent cells were passaged with 0.05% trypsin containing 1 mM EDTA, and sub-cultured and freeze down for experimental analysis.

hTERT Expression (Pre-transfection)

a. hTERT mRNA gene via Quantitative real-time PCR (qPCR) analysis

Total RNA was extracted from primary fibroblasts and cell lines by using "All prep DNA/RNA/Protein" Mini kit (Qiagen, Germany). The quality of RNA was checked on bioanalyzer by using Agilent RNA 6000 Nano kit (Agilent Technologies, Germany) and agarose gel electrophoresis. Total RNA was reverse transcribed into cDNA by using QuantiNova™ Reverse Transcription Kit (Qiagen, Germany) following manufacturer's instructions. The cDNA samples were aliquoted and stored at -20°C for future use [6]. The expression of hTERT gene in all groups

analyzed by performing PCR using primers for hTERT, hTERT forward Primer: 5´ GCATTGGAATCAGACAGCAC 3´, Reverse Primer: 5´ CCACGACGTAGTCCATGTTC 3´. GAPDH expression was amplified by using forward primer: 5´ AGCCACATCGCTCAGACAC 3´ and Reverse Primer: 5´ GCCCAATACGACCAAATCC 3´ [7]. The TaKaRa Taq HS polymerase (Takara Bio Inc, USA) was used for hTERT amplifications. All PCR products were resolved as single bands on an agarose gel (2%) through electrophoresis and visualized in bioanalytical imagining system C400 (Azure Biosystems, Inc. USA). Quantitative real-time PCR (qPCR) was performed by following the instruction provided by QuantiNova SYBR Green PCR Kit (Qiagen, Germany) and using QuantStudio 6 Flex Real-Time PCR System and QuantStudio™ Real-Time PCR Software v1.3 (Thermo Fisher Scientific). Gene expression of hTERT normalized with GAPDH to analyze the expression in study groups [8].

b. hTERT protein expression via Flow cytometer (FCM)

Expression of hTERT (human reverse transcriptase enzyme) was determined in all study groups by using flow cytometry analysis. Actively growing cells at 80% confluency were used for analysis. Culture media was removed. Cell were washed with PBS (1X) two times and proceeded for trypsination and cell count ($1x10^6$ cells). Cell were pelleted down by centrifugation and fixed by adding 100 µL fixation solution (BD Cytofix/Cytoperm™ Fixation/Permeabilization solution: BD Biosciences) for 10-15 minutes. After fixation, cells were permeabilized by adding 100 µL of a permeabilization buffer (BD Cytofix/Cytoperm™ Plus Permeabilization buffer: BD Biosciences) in the sample, mixed well and incubated at room temperature for 15 minutes. Following the incubation 2 mL of staining buffer containing 0.1% permeabilizer solution (BD Biosciences) was added to each sample and centrifuged for 5 minutes at 400 RCF. Supernatant was discarded and resuspended in 1 mL of staining buffer containing 0.1% permeabilizer solution. The cells were then

stained with hTERT antibody (NB100-317, Novus Biologicals) in staining buffer (primary antibody concentration 1:1000) for 1 hour on ice. After incubation 2 mL of staining buffer containing 0.1% permeabilizer solution (BD Biosciences) was added to each sample and centrifuged for 5 minutes at 400 RCF. Later proceeded with secondary antibody, Anti-mouse Alexa 488 (conc. 1:2000, Abcam) staining and incubation for 1 hour on ice in dark. Flow cytometry was carried out using BD Accuri™ C6 Plus Flow Cytometer (BD Biosciences) following standard procedures using BD Accuri™ C6 Plus Flow Cytometer software (BD Biosciences). Data analysis was performed using FlowJo v10 software (Ashland, USA).

c. hTERT protein expression via Immunofluorescence staining (IF)

Cells were seeded in 4 chamber slides (Thermo Fisher Scientific) at 20,000 cells per chamber and grew to medium confluency. Cells were fixed with ice cold Methanol: Acetone (1:1) at -20 °C for 10 minutes. After incubation samples were washed twice with ice cold PBS (1x) and 2 N HCl was added and incubated for 30 minutes at room temperature. HCl was aspirated and cells were rinsed twice with PBS for 5 minutes each. Permeabilization was carried out for nuclear target by incubating samples in PBS containing 0.5% Triton X-100 for 10 mins and afterwards washed with PBS three times for 5 mins. Cells were blocked by incubating in 1% BSA in PBST for 30 mins. Cells were stained with primary hTERT antibody (1:200; NB100-317, Novus Biologicals) in 1% BSA in PBST in a humidified chamber overnight at °C. After overnight incubation, solution was removed and cells were washed three times in PBS, 5 mins each wash. Cells were incubated for secondary antibody Anti-mouse Alexa 488 (1:500, Abcam) staining in 1% BSA for 1 hour at room temperature in dark. Cell were counter stained and fixed with Vectashield, containing (4, 6-diamidino-2 phenylindole) DAPI (Vector Laboratories, USA) and photographed under a 60x objective with LSM880 Airy scanning confocal microscope (Zeiss, Germany)[9].

hTERT plasmid Transfection

Plasmid propagation, isolation and analysis

E. coli GC10 strain containing eukaryotic expression plasmid (pGRN145 Features: hTERT: 4225-7623, HygB- transfection selection marker: 9216-10238, CatR: 10822-11480, AmpS: 12941-13799, PAC: 368-964, F1 ori: 11501-11685, MPSV: 3643-4119 (promoter), Ori: 12165-12747 and Bacterial selection marker: cmlR) for expressing hTERT gene, purchased from ATCC and propagated by streaking a loopful of cultures on an LB+37 µg/mL+ chloramphenicol agar plates. Bacterial colonies observed on chloramphenicol agar plates. Single colony was picked to inoculate 5mL of LB medium supplemented with chloramphenicol antibiotic. Culture were incubated for 8 hours at 37 °C while shaking at 200-250 rpm. Starter culture was diluted from 1:1000 to 1:10,000 in LB medium (50 mL bacterial culture) and incubated for 12-16 hours at 37 °C while shaking at 200-250 rpm. Bacterial culture optical density for plasmid DNA isolation was optimized (OD_{600}=2-3). Bacterial culture was harvested by centrifugation at (5000xg) for 10 mi. Supernatant was discarded and bacterial cell pellet was used immediately for plasmid DNA purification using low speed centrifuge protocol following the GeneJET plasmid Midiprep kit's instructions (catalog no. K0481, K0482, Thermo Fisher Scientific). After purification, plasmid DNA yield and quality were analyzed by using BioDrop uLITE 7141 V1.0.4 (BioDrop, UK). Confirmation of plasmid DNA was carried out by using restriction endonuclease digestion method, recommended by ATCC. Plasmid DNA was digested with XhoI and analyzed by gel electrophoresis on a 1% agarose containing SYBR safe (Invitrogen). XhoI digestion resulted in two restriction endonuclease sites at 4142 and 6160. Isolated and purified pGRN145 plasmid aliquoted and stored at -80 for later use [10]**.**

Transient transfection

hTERT Immortalized Cell System was used to develop immortalize keloid derived fibroblasts cell line by introducing hTERT gene through plasmid DNA (pGRN145) transfection (1 µg/µL) in three groups of primary keloid fibroblast (1) Peripheral (2) Middle and (3) Top Keloid fibroblasts. Cell were cultured in 6 well plates, separately until they reached 70-80% confluency and transfected with pGRN145 plasmid (ATCC MBA-141) by using *GeneXPlus* Transfection Reagent (ATCC® ACS-4004™) according to the manufacturer's instructions. Before transfection cells were counted and measured for cell density and viability. Cells were plated (7.5 $x10^4$ cells per well in 1.0 mL of complete growth medium) in 12 well plate and allowed them to grow until they reached 70-80% cell confluency. Old media was removed and replaced with 1 mL of medium containing a 4:1 mixture of DMEM: M199 and 20% FBS without antibiotic, on the day of transfection. Preparation of DNA: Transfection complexes: *GeneXPlus*, plasmid DNA and Opti-MEM I Reduced-Serum Medium, warmed to room temperature, vortexed gently to mix before use. Pipetted out 100µl Opti-MEM I Reduced-Serum Medium into a sterile microcentrifuge tube and 1.0 µL (1.0 µg/µL) plasmid DNA was added into it, mixed thoroughly with gentle pipetting. *GeneXPlus* reagent (4.0 µL) was added to the DNA mixture, mixed thoroughly by brief pipetting followed by mini centrifugation and incubation at room temperature for 15 minutes. After incubation, DNA: *GeneXPlus* transfection complex was added to the cells drop-wise at different areas of the well and distributed evenly by gentle rocking (back and forth and from side to side) the culture plates. Transfected cultures were incubated for 72 hours and transfection medium was replaced with fresh growth medium every 24 hours post transfection. Transgene expression was assayed in transfected cells by immunofluorescence staining and qPCR.

Stable transfection

In order to generate the stable cell line, Hygromycin B (HygB; antibiotic marker for transfection) was used to select antibiotic resistant parental cells (transiently transfected cells) using kill curve procedure. The parental cells were seeded at different cell count in 96-well plates in presence of antibiotic HygB (Thermo Fisher Scientific) at serial dilutions from 0 µg/mL to 1 mg/mL and incubated at 37°C. The selective medium was refreshed after every 3 days and percentage of surviving cells was observed using MTT assay. Linear regression and R square (R^2) analysis was carried out to determine the linearity of MTT assay. The minimum concentration of antibiotic was determined, that killed most cells (un-transfected host cells) within 14 days and used for selection of stable transfected cell line. Experiment was repeated to confirm the minimum selection dosage of HygB [11]**.** The resistant parent cell clone was picked for propagation and analyzed by immunofluorescence staining and confocal imagining for transfection confirmation once again. Supplementary Table 5. List of keloid site specific samples used in this study and status of immortalization and cloning. **Analysis of hTERT gene transfection:** Gene expression (mRNA) of hTERT was evaluated via Quantitative real-time PCR (qPCR) and hTERT protein expression was evaluated by flow cytometer and senescence activity assessed by senescence associated ß-galactosidase (SA-ß-gal) histochemical staining assay in all three-keloid derived transformed fibroblasts cell lines.

Defining cellular characteristics of Keloid derived immortalized fibroblasts

The stable transfected cell lines evaluated for (a) Cell viability, (b) Cell growth curve (c) Cell cycle analysis, (d) Cell Migration, (e) Cell Invasion, (f) hTERT protein expression via Immunofluorescence staining according to the methods described.

Cell viability (MTT assay)

Cell viability was analyzed by using Vybrant® MTT cell Proliferation Assay Kit (V-13154-ThermoFisher Scientific) according to the manufacturer's instructions [12].

Cell growth curve

Fibroblasts cells from all study groups were seeded ($4x10^4$ cells per well) in 12 well cell culture plates in complete medium. The culture plates were trypsinized and cell count was analyzed at fixed time intervals i: e 24, 48, 96,120 hours and 6, 7, 8, 9 and 12 days) by using automatic cell counter (Countess II FL, Life Technologies). Each time point experiment was performed in triplicate [12].

Cell cycle analysis

Cell cycle analysis (based upon quantification of total DNA content) was carried out by staining with propidium iodide (PI) and subsequently, using direct flow cytometry protocol with modifications. The experiment was performed in triplicate and results were analyzed by using De Novo FCS Express 7 Software [13].

Cell Migration

Cell migration behavior of fibroblasts was evaluated by employing *in vitro* scratch assay as described in literature. Percentage of scratch closure was measured as an indicator of cell migration activity by applying formula, C = [{A (0) - A (t)}/ A (0)] x100. In this formula, A (0) is the scratch area at 0 hour of scratch and A (t) is the scratch area at indicated time (t) point. The experiment was performed in triplicate and results were presented as graphs after analysis [14,15].

Cell Invasion

Cell invasion characteristic of fibroblasts from different groups, was studied by culturing them on transwell ECM coated plates (QCM ECMatrix Cell Invasion Assay plates, 24-well, pore size 8 µm, ECM550, Sigma-Aldrich) and subsequent imaging analysis following the assay's instructions. The experiment was performed in triplicate [16] .

Cellular senescence

Cellular senescence was investigated by using senescence associated ß-galactosidase (SA-ß-gal) histochemical staining kit (catalog no. CS00390, Sigma®). Total number of cells and blue stained cells (senescent cells, SA-ß-gal positive cells) were counted to calculate the percentage of cells expressing SA-ß- galactosidase activity and results were presented as graphs. The experiment was performed in triplicate [17].

Population doubling in transfected cell lines

The stable transfected cell lines cells were cultured ($4x10^4$ cells per well) in 12 well cell culture plates in cell growth complete media [DMEM 500 ml (Gibco), 10% FBS 50 ml, 1% AB/AM 5 ml]. After reaching 80% confluency, the culture plates were trypsinized and cell count was analyzed by using automatic cell counter (Countess II FL, Life Technologies) and subcultured with ratio of 1:2, till twelve populations. The experiment was performed in triplicate [18].

Validation of keloid derived immortalized fibroblasts cell lines.

1. Keloid site specific marker expression in keloid derived immortalized fibroblasts cell lines:

a. Analysis of protein expression via Immunofluorescence staining

Cells were seeded in 8 chamber cell culture slides (Thermo Fisher Scientific) at 2000 cells per chamber and incubated to grow to medium confluency. Cells were fixed with 4%

paraformaldehyde (PFA) for 10 minutes at room temperature. After incubation samples were washed twice with ice cold PBS (1X). Permeabilization was carried out for nuclear target by incubating samples in PBS containing 0.5% Triton X-100 and 0.25% Triton X-100 for cytoplasmic targets for 10 mins and afterwards washed with PBS three times for 5 mins. Cells were blocked by incubating in 1% BSA in PBST for 30 mins and incubated with primary Collagen I antibody (Rabbit, 1:200; NB600-408, Novus Biologicals) in 1% BSA in PBST, overnight at 4°C in humidified chamber. After incubation, solution was removed and cells were washed three times in PBS, 5 mins each wash. Cells were incubated for secondary antibody Anti-rabbit Alexa 488 antibody (1:1000, Code Number: 711-545-152, Jackson ImmunoResearch Laboratories Inc. USA) in staining buffer (1% BSA in 1x PBS) for 1 hour at room temperature in dark. Cell were counter stained and fixed with Vectashield, containing (4, 6-diamidino-2 phenylindole) DAPI (Vector Laboratories, USA) and photographed under a 60x objective with LSM880 Airy scanning confocal microscope (Zeiss, Germany) facility at University of Cape Town [9].

2. Functional evaluation: Effect of drugs on immortalized keloid derived cell lines

a. Effect of drug on apoptosis

Cells were seeded in 6 well tissue culture plates at cell count of 20,000 cells/well in complete culture medium and incubated at 37 °C in 5% CO2, to reach optimal population within 48-72 hours. Experimental and control culture were starved for 24 h in DMEM without FBS. After starvation, complete media containing Verapamil (100 µM) and Triamcinolone (100 µM) was added in experimental plate whereas control plate contained only vehicle in DMEM along with 1% FBS added in and incubate all plates for 48 hours. After incubation, FITC Annexin V staining and flow cytometric analysis used to identify apoptosis at an earlier stage for cell cultures in all study groups. FITC Annexin V Apoptosis detection carried out by following the kit's (FITC Annexin V

Apoptosis Detection Kit I, Catalog No.556547, BD Pharmingen™) instructions. Cells were washed twice with cold PBS and then resuspend in 1X Binding Buffer at a concentration of 1 x 10^6 cells/ml. 100 µl of the solution was transferred (1 x 10^5 cells) to a 5 ml culture tube and 5 µl of FITC Annexin V and 5 µl PI was added. The cells were vortexed gently and incubated for 15 min at RT (25°C) in the dark. After incubation 400 µl of 1X Binding Buffer was added and proceeded for analysis by Flow cytometer within one hour of staining. Control groups (1) unstained cells (2) cells stained with FITC Annexin V (no PI) (3) cells stained with PI (no FITC Annexin V) were used for setting up compensation and quadrants to consider groups for positive for apoptosis (FITC Annexin V positive, PI negative or FITC Annexin V positive, PI positive). Propidium Iodide (PI) is a standard flow cytometric viability probe and is used to distinguish viable from nonviable cells. Viable cells with intact membranes exclude PI, whereas the membranes of dead and damaged cells are permeable to PI. Cells that stain positive for FITC Annexin V and negative for PI are undergoing apoptosis. Cells that stain positive for both FITC Annexin V and PI are either in the end stage of apoptosis, are undergoing necrosis, or are already dead. Cells that stain negative for both FITC Annexin V and PI are alive and not undergoing measurable apoptosis [19]. De Novo FCS Express 7 Software was used for analysis.

b. Effect of drug on cell viability

Cell viability was analyzed by using Vybrant® MTT cell Proliferation Assay Kit (V-13154-ThermoFisher Scientific) according to the manufacturer’s instructions. Cells were seeded in microtiter plates at cell count of 5000 cells/well in complete culture medium and grow at 37 °C in 5% CO2, to reach optimal population within 48-72 hours. Experimental and control culture were starved for 24 h in DMEM without FBS. After starvation, complete media containing Verapamil

(100 µM) and Triamcinolone (100 µM) was added in experimental plate whereas control plate contained only vehicle in DMEM along with 1% FBS added in and incubated all plates for 48 hours. After incubation add 10 µL of 12mM MTT stock solution to each well. Negative control contained only 10µL of the MTT stock solution in 100 µL medium alone. Plates were incubated for 4 hours at 37 °C. After labelling the cells with MTT, 25 µL of medium was removed from the wells and 50 µL DMSO (Sigma) was added to each well and mixed thoroughly by pipetting and incubated at 37 °C for 10 minutes. After incubation each well mixed again and proceeded for absorbance measurement at 540 nm by using microplate reader VARIOSKAN LUX (ThermoFisher Scientific) [12].

c. Effect of drug on cell migration

Cell migration behavior of fibroblasts cells was evaluated by employing *in vitro* scratch assay. Cells were plated in 12 well cell culture plate at seeding density of 20000 cell/mL/well and allowed to grow to make a confluent monolayer. Prior to scratch, cells were starved in serum-free medium for 24 hours. After starvation, a 1000 µL pipette tip was used to create scratch in each well. Old media was removed and washed the plates twice with PBS (1X). After scratch, complete media containing drugs, Verapamil (100 µM) and Triamcinolone (100 µM) was added in experimental plate whereas control plate contained only vehicle in DMEM along with 1% FBS added and scratch zones were observed under microscope and images were taken at 0 hour from all plates. After 0-hour observation plates were placed in incubator and cell migration was monitored at 24 and 48 hours. Staining: Culture media was removed, and each well was washed two times with PBS gently. Cells were fixed with 5% PFA for 5 minutes and stained with 1% crystal violet in 2% ethanol for 20 minutes. After staining wells were washed quickly for 3 to 4 sec and dried completely by draining excess water. Scratch zones were observed under inverted

microscope (Zeiss Axiocam Vert.A1 microscope, Germany) and photographed (5x magnification) for analysis. Percentage of scratch closure was measured an indicator of cell migration activity by applying formula, C = [{A (0) - A (t)}/ A (0)] x100. In this formula, A (0) is the scratch area at 0 hour of scratch and A (t) is the scratch area at indicated time (t) point. Experiment was performed in triplicate and results were presented as graphs after analysis [14,15].

Authentication of Cell line: STR Profiling

All three cell lines were submitted to ATCC for authentication by using Short Tandem Repeat (STR) analysis. Seventeen short tandem repeat (STR) loci plus the gender determining locus, Amelogenin, were amplified and concordance criteria was set at matching matric ≥80% [20].

Cloning of Transformed cells

Successfully transformed and growing cell lines were further selected for cloning, for this purpose cell culture were trypsinized, diluted the seeding density (up to one cell per 100 mL) and seeded into 96 well plates. The culture plates were incubated at 37°C in 5% humidified CO_2 incubator. Cell growth was monitored for growth and confluency. Once the cells reached 80-90% confluency, trypsinized into 24 well tissue culture plates and subsequently subcultured into 25 cm^2 and 75 cm^2 cultured flasks for multiplication and enough cell count was stored for future use [21].

Freezing the cell line stock

Actively growing cell lines at confluency of 80-90% were selected for stock preparation and preservation. Culture medium was removed from plates and cells were washed twice with PBS (1X) and subjected to trypsinization and cell count. Cell pellet was resuspended in cell culture medium (30%) containing 60% FBS and 10% DMSO and kept in Mr. Frosty overnight and shifted to -80°C temporarily, later for long term storage, all frozen culture vials were shifted to vapour phase liquid nitrogen cabinets [21].

Supplementary Figures and Tables

Supplementary Table 1. Demographic details of study groups

Group No	Groups	Participent No	Participent ID	Sample No	Sample ID	Dermal skin tissue's sampling site	Age (Years	Gender	Ethinicity	Anatomical location
1	**Keloid skin**	1	K-035	1	KP-035	Peripheral	45	Female	African	Earlobe (Left)
				2	KM-035	Middle				
				3	KT-035	Top				
				4	KE-035	Keloid Extralesional skin tissue				
		2	K-045	5	KP-045	Peripheral	31	Female	African	Earlobe (Left)
				6	KM-045	Middle				
				7	KT-045	Top				
				8	KE-045	Keloid Extralesional skin tissue				
		3	K-046	9	KP-046	Peripheral	30	Female	African	Earlobe (Left)
				10	KM-046	Middle				
				11	KT-046	Top				
				12	KE-046	Keloid Extralesional skin tissue				
2	**Normal skin**	4	N-06	13	NF-06	Normal dermal skin	23	Female	African	Breast
		5	N-09	14	NF-09	Normal dermal skin	39	Female	African	Breast
		6	N-10	15	NF-10	Normal dermal skin	18	Female	Coloured	Breast
3	**Dermatofibro sarcoma Protuberans**	4	D-01	16	DFSP-01	Dermal skin tissue	39	Male	Coloured	Lower leg (Right)
		5	D-03	17	DFSP-03	Dermal skin tissue	40	Male	Coloured	Chest (left)
		6	D-04	18	DFSP-04	Dermal skin tissue	46	Female	Coloured	Shoulder top (Right)
4	**Fibrosarcoma**	7	FS-CL-01	19	FS-01	Dermal skin tissue	35	Male	Caucasian	Skin cancer tissue
		8	FS-CL-02	20	FS-02	Dermal skin tissue	35	Male	Caucasian	Skin cancer tissue

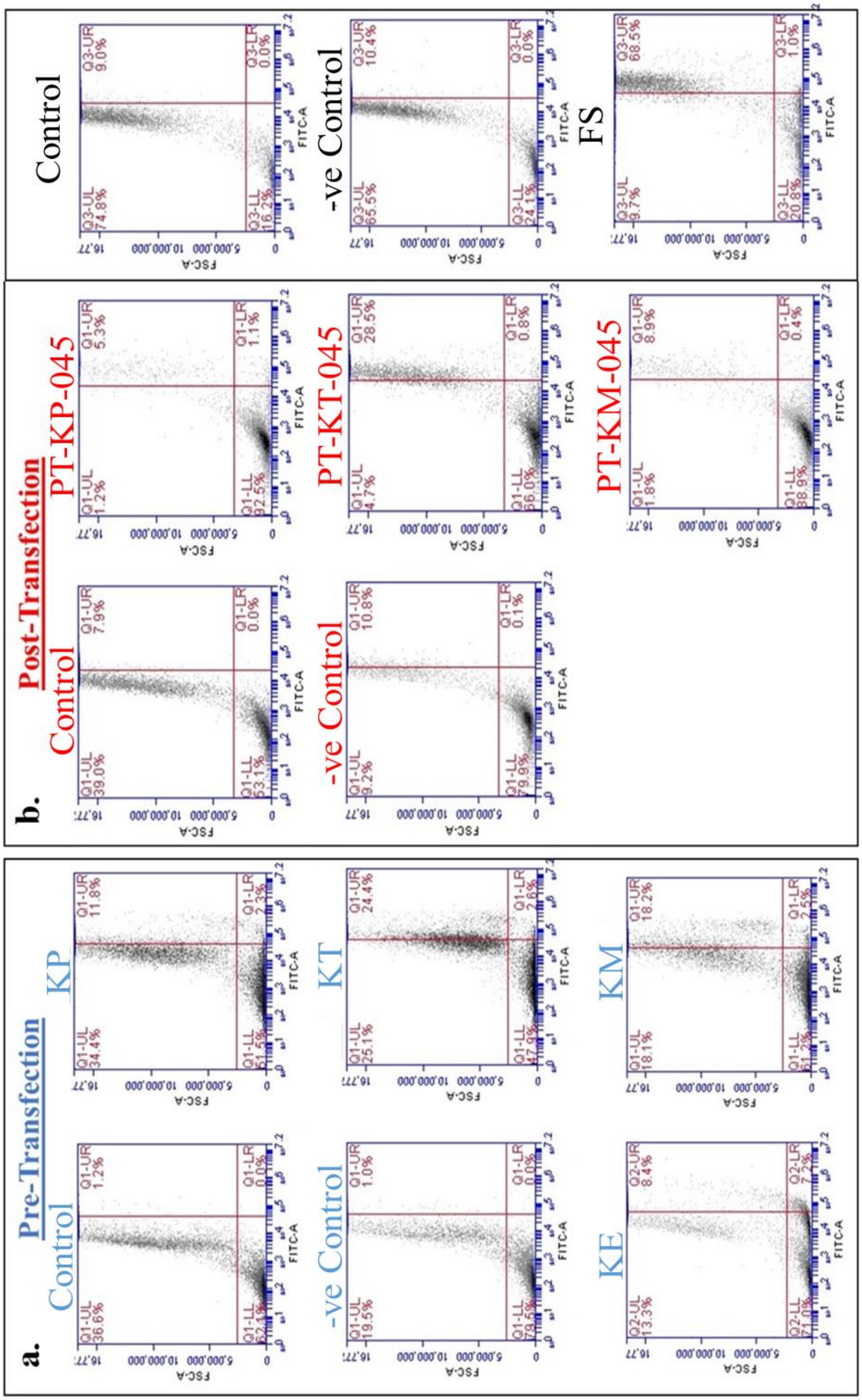
a.
Pre-Transfection
Control
KP
-ve Control
KT
KE
KM
b.
Post-Transfection
Control
PT-KP-045
-ve Control
PT-KT-045
PT-KM-045
Control
-ve Control
FS
FSC-A
FITC-A

Supplementary Figure S1. Comparative analysis of hTERT protein expression by flow cytometry in (a) primary keloid fibroblasts group (KT, KP and KM) and (b) transfected keloid fibroblasts group (PT-KP-045, PT-KT-045 and PT-KM-045) and FS as +ve control group. All four quadrant represented as, (i) Q3-UL; hTERT-FITC -ve cells, (ii) Q3-UR; hTERT-FITC +ve cells, (iii) Q3-LR; hTERT-FITC -ve cells, (iv) Q3-LL; live cell.

a.

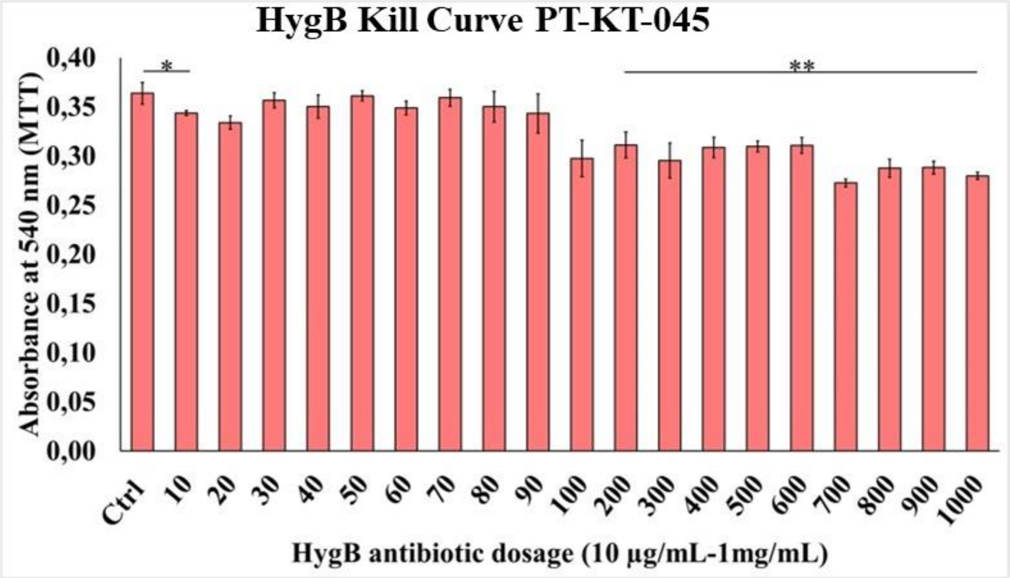
HygB Kill Curve PT-KT-045
Absorbance at 540 nm (MTT)
0,40
0,35
0,30
0,25
0,20
0,15
0,10
0,05
0,00
*
**
Ctrl 10 20 30 40 50 60 70 80 90 100 200 300 400 500 600 700 800 900 1000
HygB antibiotic dosage (10 µg/mL-1mg/mL)

b.

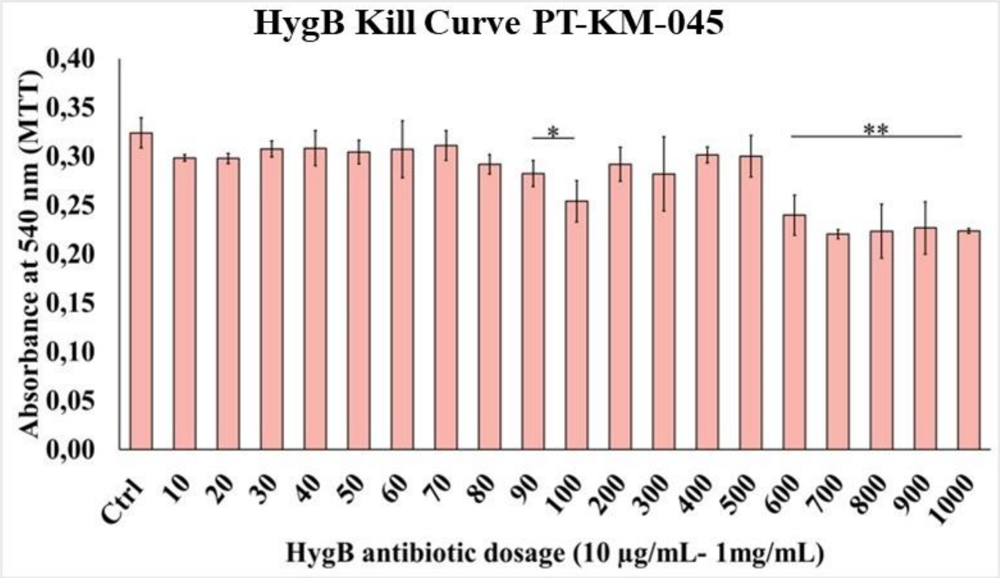
HygB Kill Curve PT-KM-045
Absorbance at 540 nm (MTT)
0,40
0,35
0,30
0,25
0,20
0,15
0,10
0,05
0,00
*
**
Ctrl 10 20 30 40 50 60 70 80 90 100 200 300 400 500 600 700 800 900 1000
HygB antibiotic dosage (10 µg/mL- 1mg/mL)

c.

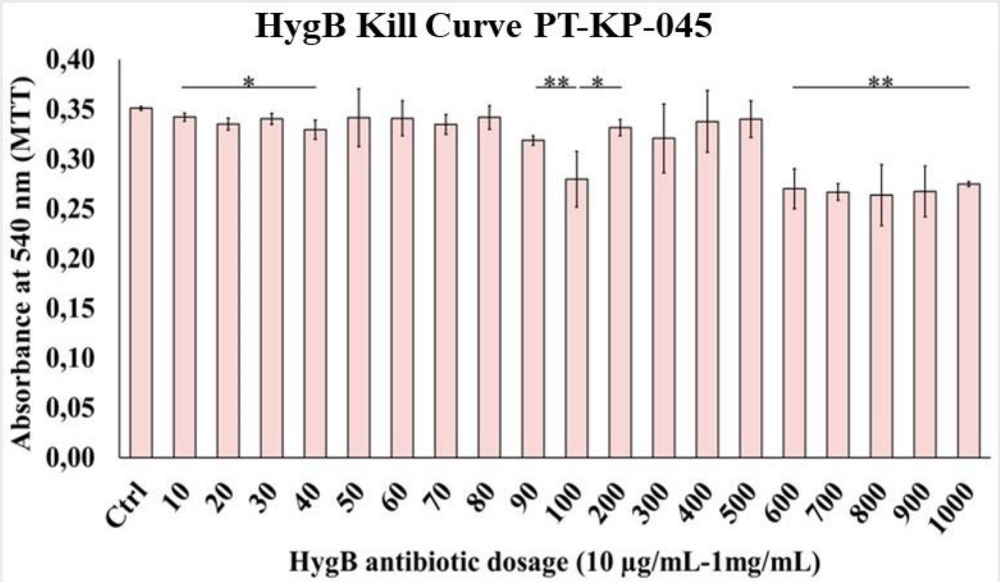
HygB Kill Curve PT-KP-045
Absorbance at 540 nm (MTT)
0,40
0,35
0,30
0,25
0,20
0,15
0,10
0,05
0,00
*
** *
**
Ctrl 10 20 30 40 50 60 70 80 90 100 200 300 400 500 600 700 800 900 1000
HygB antibiotic dosage (10 µg/mL-1mg/mL)

Supplementary Figure S2. Stable transfection. Antibiotic resistance-based selection (HygB kill curve), post-transfection selection of Hygromycin B resistant cells in all three cell lines **(a)** PT-KT-045, **(b)** PT-KM-045, **(c)** PT-KP-045, at concentration ranges from 0 µg/mL-1000 µg/mL) at 48-hour antibiotic treatment and cell viability evaluated via MTT assay.

Supplementary Table 2. List of keloid site specific samples used in this study and status of immortalization and cloning.

S. No	Keloid sample	Keloid tissue site	Sample labels/ Abbreviations*	Primary cell line established	Primary keloid fibroblast cell lines	Immortalisation via hTERT plasmid (pGRN145 (ATCC® MBA141™) transfection	Cloning
1.	KD-UP-035	Middle	KD-UP-K-M-035	Yes	1	PT-KM-035	S
		Top	KD-UP-K-T-035	Yes	2	PT-KT-035	NS
		Peripheral	KD-UP-K-P-035	Yes	3	PT-KP-035	NS
2.	KD-UP-045	Middle	KD-UP-K-M-045	Yes	4	PT-KM-045	S
		Top	KD-UP-K-T-045	Yes	5	PT-KT-045	S
		Peripheral	KD-UP-K-P-045	Yes	6	PT-KP-045	S
3.	KD-UP-046	Middle	KD-UP-K-M-046	Yes	7	PT-KM-046	NS
		Top	KD-UP-K-T-046	Yes	8	PT-KT-046	NS
		Peripheral	KD-UP-K-P-046	Yes	9	PT-KP-046	NS

S= Stable transfection, NS= Non-stable transfection

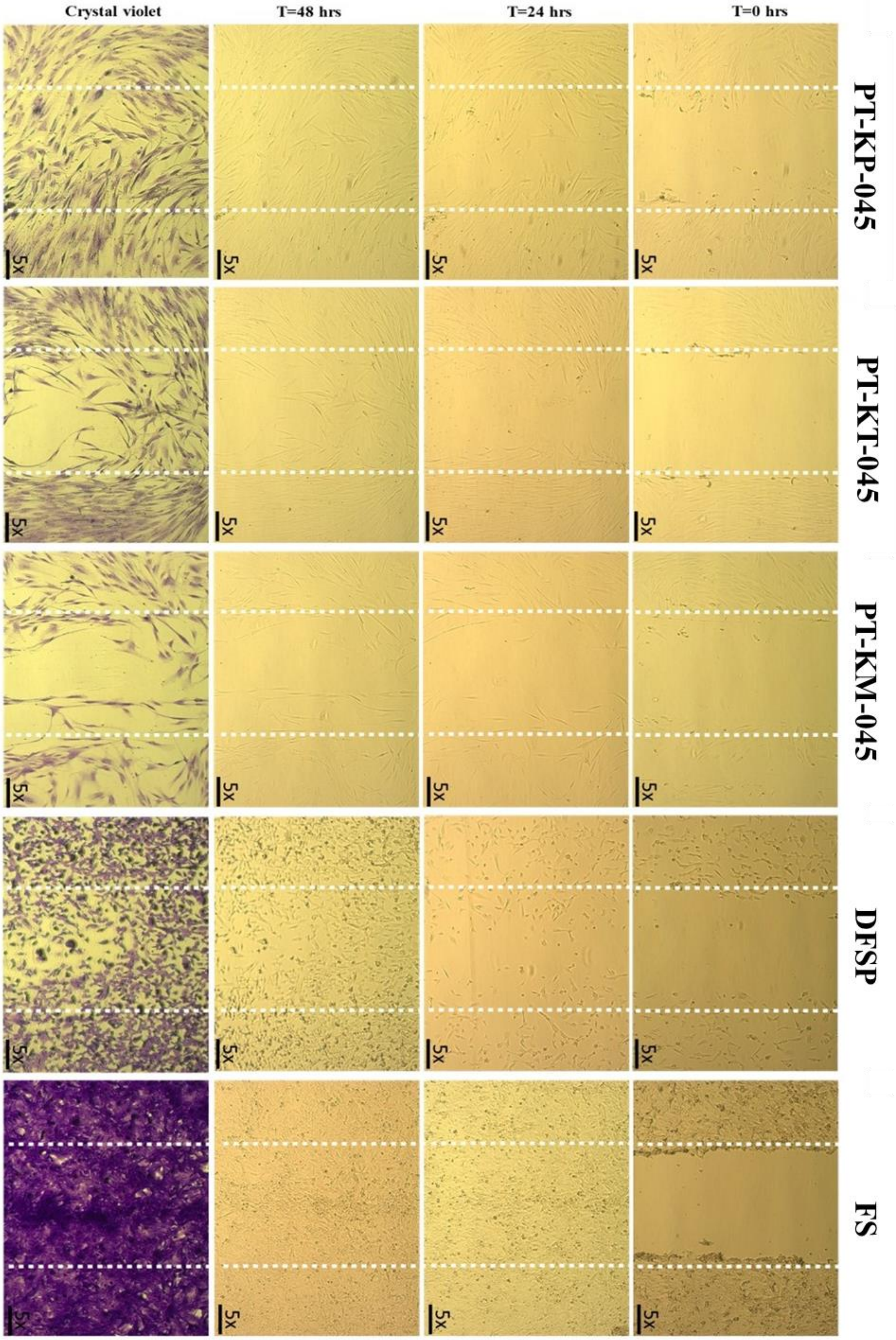
Crystal violet
T=48 hrs
T=24 hrs
T=0 hrs
PT-KP-045
PT-KT-045
PT-KM-045
DFSP
FS
5x

Supplementary Figure S3. Comparative cell migration evaluated in transfected keloid fibroblasts group (PT-KP-045, PT-KT-045 and PT-KM-045) and DFSP and FS (+ve control group) by employing *in vitro* scratch assay and cell migration observed towards scratch zone at 24 and 48 hours' time points. At end point cell were stained with crystal violet stain and photographed. Scratch margin marked with white dotted line. Image scale 5x.

Supporting Information References

1. Bayat A, McGrouther DA, Ferguson MW. Skin scarring. *BMJ (Clinical research ed.)* 2003; 326(7380):88–92.
2. Bayat A, Arscott G, Ollier WER, Fergusond MWJ, McGrouthera DA. Description of site-specific morphology of keloid phenotypes in an Afrocaribbean population. J Plast Reconstr Aesthet Surg 2004; 57:122–133.
3. Bayat A, Arscott G, Ollier WER, Mc Grouther DA, Ferguson MWJ. Keloid disease: clinical relevance of single versus multiple site scars. Br J Plast Surg 2005; 58(1):28–37.
4. Kelly AP, Bayat A. Keloids. Taylor and Kelly's Dermatology for Skin of Color, Chapter 33: 2e. Kelly A, & Taylor S.C., & Lim H.W., & Serrano A (Eds.), McGraw-Hill. 2016.
5. Limandjaja GC, van den Broek LJ, Waaijman T, Breetveld M, Monstrey S, Scheper RJ. Reconstructed human keloid models show heterogeneity within keloid scars. Arch Dermatol Res 2018; 310:815–826.
6. Gabet A-S, Accardi R, Bellopede A, Popp S, Boukamp P, Sylla BS, et al. Impairment of the telomere/telomerase system and genomic instability are associated with keratinocyte immortalization induced by the skin human papillomavirus type 38. FASEB J 2008; 624:22.
7. Arenas-Hernandez M, Vega-Sanchez R. Housekeeping gene expression stability in reproductive tissues after mitogen stimulation. BMC Res Notes 2013; 6:285.

8. Saleh S, Lam A K-Y, Yik-HongHo. Real-time PCR quantification of human telomerase reverse transcriptase (hTERT) in colorectal cancer. Pathol 2008; 40(1):25–30.
9. Sadiq A, Shah A, Jeschke MG, Belo C, Hayat MQ, Murad S, et al. The role of serotonin during skin healing in post-thermal injury. Int J Mol Sci 2018; 19(4):1034.
10. Lanza AM, Kim DS, Alper HS. Evaluating the influence of selection markers on obtaining selected pools and stable cell lines in human cells. Biotechnol J 2013; 8(7):811–21.
11. Delrue I, Pan Q, Baczmanska AK, Callens BW, Verdoodt LLM. Determination of the selection capacity of antibiotics for gene selection. Biotechnol J 2018; 13(8): e1700747.
12. Giugliano G, Pasquali D, Notaro A, Brongo S, Nicoletti G, D'Andrea F, et al. Verapamil inhibits interleukin-6 and vascular endothelial growth factor production in primary cultures of keloid fibroblasts. J. Plast.Reconstr. Aesthet. Surg 2003; 56: 804–809.
13. Blasi T, Hennig H, Summers HD, Theis FJ, Cerveira J, Patterson JO, et al. Label-free cell cycle analysis for high-throughput imaging flow cytometry. Nat Commun 2016; 7: 10256.
14. Chen Y, Lu B, Yang Q, Fearns C, Yates JR, Lee JD. Combined integrin phosphoproteomic analyses and siRNA-based functional screening identified key regulators for cancer cell adhesion and migration. Cancer Res 2009:69(8):3713–3720.
15. Barczak W, Sobecka A, Golusinski P, Masternak MM, Rubis B, Suchorska WM, et al. hTERT gene knockdown enhances response to radio- and chemotherapy in head and neck cancer cell lines through a DNA damage pathway modification. Sci Rep 2018; 8:5949.
16. Xia L, Huang W, Bellani M, Seidman MM, Wu K, Fan D, et al. CHD4 has oncogenic functions in initiating and maintaining epigenetic suppression of multiple tumour suppressor genes. Cancer Cell 2017; 31:653–668.
17. Itahana K, Itahana Y, Dimri GPD. Colorimetric detection of Senescence-Associated β Galactosidase. Methods Mol Biol 2013; 965:143–156.

18. Teng Z, Yoshida T, Okabe M, Toda A, Higuchi O, Nogami M, et al. Establishment of immortalized human amniotic mesenchymal stem cells. Cell Transplant 2013; 22(2):267–78.

19. Wlodkowic D, Skommer J, Darzynkiewicz Z. Flow Cytometry-Based Apoptosis Detection. In: Erhardt P., Toth A. (eds) Apoptosis. Methods Mol Biol (Methods and Protocols) 2009; vol 559. Humana Press, Totowa, NJ.

20. Almeida JL, Cole KD, Plant AL. Standards for cell line authentication and beyond. PLoS Biol 2016; 14(6):e1002476.

21. Crameri G, Todd S, Grimley S, McEachern JA, Marsh GA, Smith C, et al. Establishment, immortalisation and characterisation of Pteropid Bat cell lines. PLoS ONE 2009; 4(12): e8266.
